# Temperature-dependent life history and transcriptomic responses in heat-tolerant versus heat-sensitive *Brachionus* rotifers

**DOI:** 10.1038/s41598-020-70173-0

**Published:** 2020-08-06

**Authors:** Sofia Paraskevopoulou, Alice B. Dennis, Guntram Weithoff, Ralph Tiedemann

**Affiliations:** 1 Unit of Evolutionary Biology and Systematic Zoology, Institute of Biochemistry and Biology, University of Potsdam, Potsdam, Germany; 2 Unit of Ecology and Ecosystem Modelling, Institute of Biochemistry and Biology, University of Potsdam, Potsdam, Germany; 3 Berlin-Brandenburg Institute of Advanced Biodiversity Research (BBIB), 14195 Berlin, Germany

**Keywords:** Evolution, Ecology

## Abstract

Thermal stress response is an essential physiological trait that determines occurrence and temporal succession in nature, including response to climate change. We compared temperature-related demography in closely related heat-tolerant and heat-sensitive *Brachionus* rotifer species. We found significant differences in heat response, with the heat-sensitive species adopting a strategy of long survival and low population growth, while the heat-tolerant followed the opposite strategy. In both species, we examined the genetic basis of physiological variation by comparing gene expression across increasing temperatures. Comparative transcriptomic analyses identified shared and opposing responses to heat. Interestingly, expression of heat shock proteins (*hsps*) was strikingly different in the two species and mirrored differences in population growth rates, showing that *hsp* genes are likely a key component of a species’ adaptation to different temperatures. Temperature induction caused opposing patterns of expression in further functional categories including energy, carbohydrate and lipid metabolism, and in genes related to ribosomal proteins. In the heat-sensitive species, elevated temperatures caused up-regulation of genes related to meiosis induction and post-translational histone modifications. This work demonstrates the sweeping reorganizations of biological functions that accompany temperature adaptation in these two species and reveals potential molecular mechanisms that might be activated for adaptation to global warming.

## Introduction

On a global scale, a species’ occurrence is related to its tolerance of a particular range of environmental parameters such as temperature, salinity and precipitation. In aquatic ecosystems, temperature has a profound impact on an organism’s survival and performance, and can affect species abundance, spatio-temporal distribution, habitat colonization and species interactions^1–3^. There is large variation in the thermal tolerance among aquatic taxa. Many species can tolerate a broad range of temperatures, while others have specific and narrow temperature limits^4,5^. Importantly, this can impact temporal occurrence and temperature-dependent seasonal succession has been well documented among genetically similar species that might have evolved species-specific temperature specializations^6–9^. Therefore, knowledge of thermal boundaries is essential to understand how species have adapted to their environment and how they may respond to climate change.

Zooplankton represent an important component of aquatic ecosystems, as they transfer organic compounds and energy from primary producers (e.g. phytoplankton) to higher trophic levels^10^. Among zooplankton, monogonont rotifers are of particular interest because of their high, often cryptic, diversity, their frequent adaptation to specific environmental conditions, and high dispersal capability^11,12^. Species complexes formerly assumed to be ubiquitous generalists have been found to comprise cryptic species adapted to specific ecological conditions regarding temperature, habitat type, or salinity^13–15^. As their dispersal capabilities can be large, distribution and diversification seem less dependent on geographical barriers and historical factors, suggesting that ecological specialization is more likely to drive speciation^13–16^. As evidence of specialization, co-occurrence of differentially adapted, closely related species in a single locality is a common phenomenon in rotifers^6,7,17^. In these cases, morphologically similar species might have evolved different ecological specialties to reduce competition over resources in space or time^15,18,19^.

The best studied freshwater monogonont rotifer is the *Brachionus calyciflorus* species complex that has recently been resolved to four different species using integrative taxonomy: *Brachionus calyciflorus* sensu stricto (s.s.), *Brachionus fernandoi*, *Brachionus dorcas,* and *Brachionus elevatus*^20,21^. The species of this complex exhibit temporal succession, and their occurrences have been related to temperature in several studies^7,8,17,22^. More specifically, temperature constraints were shown to affect the temporal occurrence and abundance of *B. calyciflorus* cryptic species in different habitats in China^7,17^. *Brachionus calyciflorus *s.s. occurred mainly in summer at a maximum temperature of 32 °C^7^ while *B. fernandoi* mainly during winter and spring under a maximum temperature of 19 °C^7^. Both species generally exhibit a non-overlapping seasonal occurrence during summer and winter respectively, however, in November they co-occurred with *B. fernandoi* outnumbering *B. calyciflorus* s.s. in abundance^7^. Our previous comparative laboratory study on heat tolerance between different clones belonging to *B. calyciflorus* s.s. and *B. fernandoi* species has shown higher heat-tolerance of the former as compared to the latter^23^. In this study, acute heat-stress was imposed and the Critical Thermal Maximum (CT_*max*_) was estimated as a proxy for survival. Heat resistance was species, but not clone, specific with values of CT_*max*_ varying 10 °C between the species^23^. Therefore, we confirmed that temperature tolerance likely plays a role in their temporal distribution^23^.

Understanding the ability of species to adapt to environmental change necessitates knowledge of the genomic basis of the relevant adaptations. The rapid development of high-throughput sequencing technologies and whole transcriptome profiling (RNA-seq) has enabled a deeper investigation of adaptive and functional variation in model and non-model species^24^. Regulation of gene expression is an essential mechanism underlying physiological robustness as well as phenotypic plasticity^25^. Since selection acts on the sequence itself (DNA), but also on expression, transcriptome data are particularly useful in revealing the genetic basis of adaptation, i.e., genes contributing to fitness by either structural (non-synonymous substitutions) or expression differences. In recently diverged snake species for example, significant expression differentiation was identified with little and non-coding sequence variation across populations, demonstrating that expression differentiation was the exclusive genetic basis of polygenic adaptation^26^. By profiling transcriptional changes induced by temperature, it is possible to identify the gene regions or pathways that are likely to be targets of temperature-driven selection^27,28^.

Rotifers have been used as model organisms to understand complex processes such as the evolution of sex, aging, and stress responses to toxic compounds^29–33^. However, transcription studies on temperature response have often focused mainly on genes involved in generation of oxidative stress and genes encoding for heat shock proteins (*hsps*)^34–37^. Heat shock proteins are divided into several groups (families) of different molecular weights (kDa): e.g. *hsp90, hsp70, hsp60, hsp40*, and small proteins. *hsp70s*, in combination with other proteins, play a vital role in stress tolerance and survival under adverse conditions. They reduce accumulation of peptide aggregates and promote the correct folding of newly synthesized proteins^38^. *hsp90s* also play a major role in stress tolerance, mainly by removing incorrectly folded proteins. Furthermore, they regulate the activity of other proteins (e.g., kinases) and stabilize the cytoskeleton^39^. Induction of *hsp* genes is an evolutionary old and conserved mechanism, and is described from prokaryotes to higher eukaryotes^40^. However, the specific genes involved and the conditions of induction vary among taxa^41^. In rotifers, particularly *Brachionus* species, members of the *hsp70* and *hsp40* families increase heat shock survival, suggesting that there may be coordination among heat shock proteins in which *hsp40* works synergistically to regulate *hsp70*’s activity (as shown in *B. manjavacas*^37^).

To investigate the marked variation in thermal tolerance between two closely related species in the former *B. calyciflorus* species complex, we compared life-history demography and gene expression under mild to high temperature conditions. We used life-table experiments to examine survival, fecundity, and population growth rate differences between the two species. We collected transcriptomic data (RNA-seq) to examine the genetic basis of physiological response and its difference between the two species. The mechanisms we identify are important to understand how physiology determines species’ temporal distribution, and how this might be affected by different scenarios of climate change.

## Materials and methods

### Rotifer culture and life table experimental conditions

In a previous laboratory study, we quantified acute heat response of different clones belonging to *B. calyciflorus* s.s. (10 clones) and *B. fernandoi* (5 clones)^23^. Thermal tolerance was estimated as a bi-dimensional phenotypic trait affected by both the intensity and duration of the heat by measuring time to incapacitation which is considered a proxy for survival^42^. Cross-species differences were revealed in the maximum temperature (CT_*max*_) that the species were able to tolerate^23^. For the purpose of this study we selected two asexually reproducing clones with known differences in the maximum temperature they can sustain (CT_*max*_) under acute heat exposure^23^. One clone represented the heat-tolerant *B. calyciflorus *s.s. (clone IGB; CT_*max*_ = 43.18 °C) and the other one the heat-sensitive *B. fernandoi* (clone A10; CT_*max*_ = 38.49 °C)^23^. Both clones originate from Northern Germany and were reared under laboratory conditions for more than 10 years. Species classification was previously confirmed by amplifying a portion of the ITS1 genetic marker^23^. Stock cultures were maintained in WC medium^43^ at 20 °C under a 16:8 light:dark photoperiod. A food combination of two algae species, *Monoraphidium minutum* (Culture collection Göttingen, strain SAG-243-1) and *Cryptomonas* sp. (Culture collection Göttingen, strain SAG-26-80), was provided weekly.

Before starting the life table experiments, cultures were exposed to a period of gradual acclimatization by increasing the temperature 2 °C every 2 days until reaching the experimental temperature. Because of this, the acclimation period varied among cultures, with the longest adaptation period (2 weeks) for the highest temperature (32 °C). After reaching the experimental temperature, we maintained the rotifer cultures for one week (at least two generations) before starting the experiment to reduce potential maternal effects. Food was supplied *ad libitum* daily. For *B. calyciflorus* s.s., experiments were conducted at four temperatures (20 °C, 23 °C, 26 °C, 32 °C), while for *B. fernandoi* three temperature assays (20 °C, 23 °C, 26 °C) were used. We tried several times to acclimate *B. fernandoi* to 32 °C, but high mortality always led to culture collapse before the initiation of the experiment.

During the experiments single females bearing a subitaneous, asexual egg were isolated from the culture, placed in a microtitre well, and inspected thoroughly for hatched neonates. Life-table experiments were started by introducing one neonate into a new well to 1 ml of algal suspension composed of *Monoraphidium minutum* (5 × 10^5^ cells/ml) and *Cryptomonas* sp. (2 × 10^4^ cells/ml), to avoid food limitation. For each temperature and species, at least 24 individuals were recorded. Survival and reproduction were recorded every 12 h and any newly hatched neonates were removed. Every 24 h the maternal individuals were transferred into a new well with fresh food suspension. The experiment was conducted in the dark and continued until all individuals of each cohort died^44,45^.

### Computations and statistical analysis

The variables of average life span (survival), age-specific survival (lx) and age-specific fecundity (mx) were estimated for each species and temperature assay (x was defined as the age interval in days, lx as the proportion of surviving individuals at the beginning of the age interval and mx as the number of offspring produced per female alive from the start until the end of any age interval^46^. The intrinsic rate of population increase (*r*) was also estimated from these data using Lotka’s equation (Eq. 1)^47^:

1 $$r = \sum e^{ - rx} *lx\; \, mx \, = 1$$

Kaplan–Meier survival curves were calculated to compare survival across species and temperatures. Differences between survival functions were analyzed pairwise using a log-rank test. To further estimate the effect of species, temperature, and their interaction on lifespan and fecundity, we used generalized linear models (GLMs). We selected the best-fitting model based on the AIC criterion^48^. To compare fecundity among the species and temperature treatments we used the non-parametric Kruskal–Wallis (K–W) one-way analysis of variance. For pairwise comparisons the pairwise Wilcox test was used with a Bonferroni correction. Non parametric tests were used for comparisons, as all of the assumptions to perform a parametric Analysis of Variance (ANOVA; normal distribution of the data, normal distribution of the residuals, and homoscedasticity) were violated. To compare the intrinsic rate of increase (*r*), the 95% confidence interval was estimated via bootstrapping with 199 iterations^49^ using a custom n R script. All statistical analyses were performed using R 3.4.1^50^.

### Sample cultivation, collection, and RNA isolation

**Figure 1:**
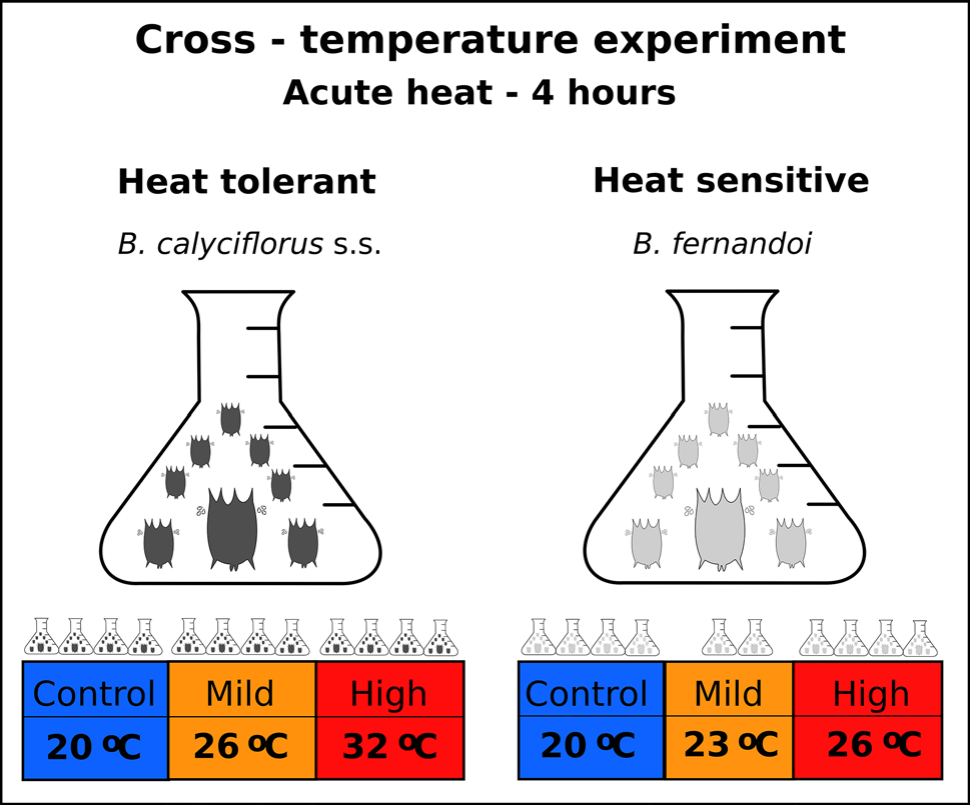
Experimental design for transcriptomic responses to heat exposure between heat-tolerant and the heat-sensitive *Brachionus* species. Temperature regimes were chosen to represent control (20 °C), mild (26/23 °C) and high (32/26 °C) heat treatment for each species. (Figure made in Inkscape v.1.0; https://inkscape.org/).

Samples for RNA-seq were first cultivated as batch cultures in 1 l glass bottles containing WC medium at 20 °C under 16:8 light:dark photoperiod. The same two algae combination was provided ad libitum every 2 days for 2 weeks before the RNA-seq experiment to allow for substantial population growth. For each species and temperature, we sub-sampled the initial stock into new flasks to create four replicates of 200 ml each, containing approximately 1,000 individuals. We placed the flasks into water-baths adjusted to the experimental temperature and heat-exposed the rotifers for 4 h. Experimental temperatures were selected according to the life-table results in order to represent control (20 °C; temperature in which both clones were acclimated), mild heat treatment (23 °C for *B. fernandoi;* 26 °C for *B. calyciflorus* s.s.; intermediate temperature between control and high heat exposure), and high heat treatment (26 °C for *B. fernandoi* and 32 °C for *B. calyciflorus* s.s.; temperature in which the population growth rate starts to decline) (Fig. 1). In addition, preliminary experiments during which temperature was increased 2 °C every 4 days showed that 32 °C and 26 °C represent the threshold temperature above which population growth rate became negative, for *B calyciflorus* s.s. and *B. fernandoi,* respectively. For specimen collection we filtered each replicate (200 ml) through a 30 μm sieve, re-suspended what remained on the filter in WC medium, and centrifuged it at 2,000×*g* for 10 min to pellet phytoplankton and other debris, before transferring the rotifers (remaining in the supernatant) into 1 ml of TRIzol LS and storing them at − 80 °C until RNA extraction. For RNA extraction, we used four replicates from each temperature and species with the exception of *B. fernandoi* at mild heat, where two replicates were used. Each replicate, comprised approximately of 1,000 individuals, was reared in independent flasks of 200 ml.

Samples in TRIzol LS, after having been homogenized using a Tissue Lyzer (4 min, 50 Hz), were incubated overnight at room temperature. A total of 500 μl of chloroform was added to each sample and samples were centrifuged for 15 min at 4 °C to facilitate phase separation^51^. The colorless, upper aqueous phase was transferred into an RNeasy Mini Kit column (Qiagen, Germany) and proceeded to RNA precipitation according to the manufacturer’s instructions. Total RNA concentration was estimated using a NanoDrop 1000 spectrophotometer (ThermoFischer Scientific, Germany). Quality of total RNA was examined using Agilent Bioanalyzer 2100 (Agilent Technologies, USA).

For transcriptomic library preparation, enrichment of mRNA from total RNA (3 μg) was performed with poly (A) capture using NEXTflex Poly (A) Beads. Strand-specific libraries were constructed using NEXTflex Rapid Directional RNA-Seq Kit (Bioo Scientific, USA) according to manufacturer’s instructions. Final elution was performed in 16 μl of elution buffer and a PCR amplification of 14 cycles was performed^51^. Libraries were quantified using Qubit dsDNA HS Assay Kit (Invitrogen, Germany) and quality control was performed using Agilent Bioanalyzer 2100 (Agilent Technologies, USA).

### Transcriptome sequencing, assembly and annotation

Libraries were sequenced as 150 bp paired-end (PE) reads using an Illumina HiSeq 4000 sequencing system, performed by Novogene (Hong Kong, China). Raw data have been deposited in the NCBI Short Read Archive under the accessions numbers (SRA: SRR10426055-76). Adapter sequences were trimmed and low quality reads were filtered using a 5 bp sliding window with a mean quality threshold of 20 and minimum read length of 36 bp using Trimmomatic v0.36^52^. Read quality (before and after quality-filtering) was assessed using FastQC v0.11.5^53^.

Processed reads were assembled de novo with Trinity v.2.5.1^54^. Two separate transcriptome assemblies were created for *B. calyciflorus* s.s. and *B. fernandoi*, using all reads generated for each respective species. For the assembly and further read quantification we did not utilize the recently produced *B. calyciflorus* s.s. genome as this would introduce a bias, since the *B. fernandoi* reads would not map as well to the reference as those from *B. calyciflorus* s.s.. We, however, used the genome assembly to filter out contaminating algae sequences. Since each replicate contained rotifer individuals and algae from the culture medium, removing contaminants (i.e. reads belonging to algae) was essential before any further analysis. To filter out contamination, we used a custom perl script which, using the blastn algorithm (ncbi-blast-2.6.0^55^), assigns all contigs either to a local algae database (*Monoraphidium minutum*, *Chlamydomonas reinhardtii* and, *Cryptomonas* sp) or to the respective *B. calyciflorus *s.s. genome assembly^56^. Transcripts were only assigned as of rotifer origin when the top hit was to the *B. calyciflorus* s.s. genome and the bit-score gain over matches to the next species was > 100. The same custom script was further used to remove ribosomal RNA reads by performing a blastn search to a local database consisting of 18S and 28S sequences of *Brachionus* species downloaded from NCBI.

### Identification of differentially expressed genes and pathways

Gene-level quantification estimates produced by RSEM^57^ were imported into R/Bioconductor with the tximport package. The tximport package produces count matrices from gene-level quantification files with effective gene length taken into account^58^. To detect differential gene expression, we passed the estimated count matrices from tximport to DESeq2^59^ and analyzed the two species separately. To build the model for the differential expression analysis we removed low count genes (< 10) and genes that were not present in at least 2 replicates. To examine intra-species temperature specific expression pattern in *B. calyciflorus* s.s., we performed pairwise contrasts within the model in the following combinations: 20 vs. 26 °C (control vs. mild heat), 20 vs. 32 °C (control vs. high heat), and 26 vs. 32 °C (mild vs. high heat) (Fig. 1). For *B. fernandoi* similarly we performed pairwise comparisons in the following combinations: 20 vs. 23 °C (control vs. mild heat), 20 vs. 26 °C (control vs. high heat), and 23 vs. 26 °C (mild vs. high heat) (Fig. 1). We used a false discovery rate (FDR) threshold of 0.05 to correct for multiple testing. All differentially expressed genes (DEGs) were annotated against the NCBI non-redundant (*nr*) database using blastx (e-value cutoff 1e^−10^). We assessed overall temperature-dependent patterns of expression by plotting a two-dimensional principal component analysis (PCA) of log-transformed counts for each species separately. Heatmaps representing differential expressed genes were constructed by using the Heatmapper program^60^.

We further categorized differential expression at the gene-pathway level using the online Kyoto Encyclopedia of Genes and Genomes (KEGG) automatic server for KEGG pathway analysis^61–63^, which clusters genes based on their association in biochemical pathways. To estimate whole KEGG pathway expression, genes belonging to the same KEGG pathway were clustered together and a differential pathway expression analysis was performed. Pathways were considered differentially expressed at a false discovery rate (FDR) below 0.05. Analyses and visualization was performed using the “gage”^64^, “clusterProfiler”^65^, “pathview”^66^, and “ggplot2”^67^ R packages. Heatmaps representing differentially expressed KEGG pathways were constructed in R^50^.

To capture similar or contrasting patterns of expression among the two species, orthologous genes were identified using OrthoFinder^68^. From this, we compared expression of orthologous genes between *B. fernandoi* and *B. calyciflorous* using Clust^69^*.* In Clust*,* gene clusters (groups) are identified that are consistently co-expressed (well-correlated) in both shared and contrasting patterns between species. Within this, we chose patterns that were biologically meaningful for further analysis. These groups/clusters were checked for functional enrichment in any KEGG pathway, using a Fisher Exact Test and correcting for false positives (FDR = 0.05).

## Results

### Life history responses to elevated temperatures

**Figure 2:**
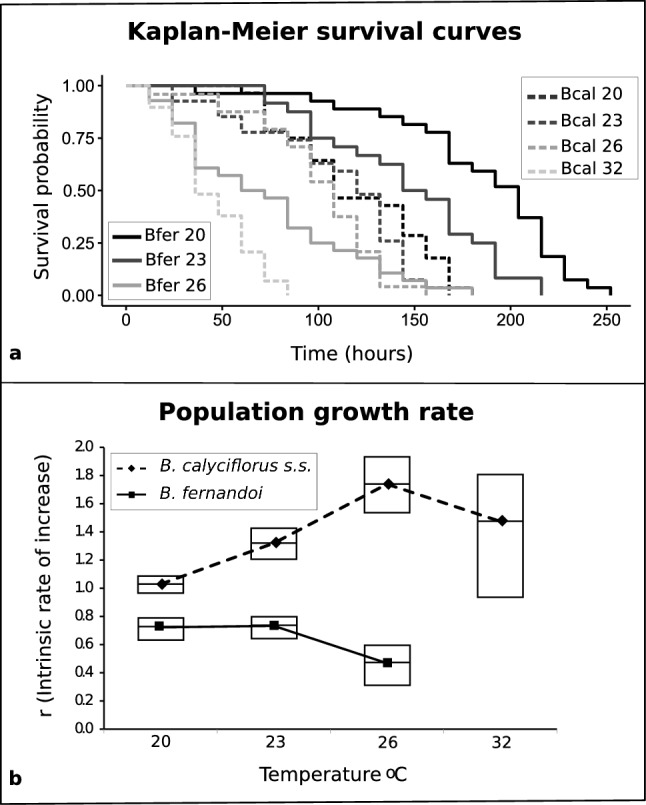
Kaplan–Meier survival curves (**a**), and population growth rate/*r* (**b**) for the heat-tolerant *B. calyciflorus* s.s. (Bcal) and the heat-sensitive *B. fernandoi* (Bfer) under different temperature conditions. Solid lines denote responses of *B. fernandoi* to different temperatures (20 °C, 23 °C, 26 °C), while dashed lines represent responses of *B. calyciflorus* s.s (20 °C, 23 °C, 26 °C, 32 °C). Boxes denote the 95% confidence intervals estimated via bootstrap with 199 iterations. (Figure produced by using R 3.4.1^50^).

Survival, fecundity and the resulting population growth rate of both species were strongly affected by temperature. For both species, survival was significantly reduced from control to mild and from mild to high heat (Fig. 2a, all p-values available in Supplementary Table 1). Cross-species survival comparisons revealed significant differences at 20 °C (p < 0.001) and 23 °C (p = 0.008), with *B. fernandoi* surviving longer than *B. calyciflorus* s.s (Fig. 2a, Supplementary Table 1). Fecundity analysis with the K–W test revealed that both *B. calyciflorus* s.s. and *B. fernandoi* had a significant lower fecundity at the highest imposed heat (*B. calyciflorus* s.s.: 20 °C vs*.* 32 °C, p < 0.001; 23 °C vs. 32 °C, p = 0.001; 26 °C vs. 32 °C, p = 0.002; *B. fernandoi*: 20 °C vs. 26 °C, p < 0.001; 23 °C vs. 26 °C, p = 0.004, Supplementary Fig. 1A, Supplementary Table 2). Across the two species, significant differences were observed at 26 °C, in which the heat-tolerant *B. calyciflorus* s.s. had higher fecundity than the heat-sensitive *B. fernandoi* (Bcal26 vs. Bfer26, p < 0.001, Supplementary Fig. 1A, Supplementary Table 2). Generalized linear models revealed that both survival and fecundity were significantly dependent on temperature (GLM: survival, p < 0.001; fecundity, p < 0.001), species (GLM: survival, p < 0.001; fecundity, p = 0.006), and their interaction (GLM: survival, p < 0.001; fecundity, p = 0.001) (Supplementary Figs. 1B, 1C).

The intrinsic rate of population increase (*r*) was above zero for both species, indicating a positive growth rate at all tested temperatures. However, *r* was always higher for the heat-tolerant *B. calyciflorus* s.s. than for the heat-sensitive *B. fernandoi* (Fig. 2b)*.* For *B. calycifloryus* s.s, *r* increased with the increase of temperature until its maximum at 26 °C. In contrast, *r* remained constant in *B. fernandoi* from 20 °C to 23 °C, and decreased at 26 °C.

### Comparative transcriptomics and cross-temperature differential gene expression

Sequencing of the *B. calyciflorus* s.s. and *B. fernandoi* transcriptomes generated 670,242,336 and 358,527,278 quality-filtered PE reads, respectively, with approximately equal numbers of reads among libraries (Supplementary Table 3). Information about the de novo assemblies, assembled unigenes, and KEGG KO term assignment can be found in Table 1.

**Table 1:** Summary statistics of the de novo assemblies of the *Brachionus calyciflorus* s.s. and *B. fernandoi* transcriptomes.

Transcriptome assembly statistics	*B. calyciflorus* s.s	*B. fernandoi*
# Raw reads (n)	798,693,466	430,046,346
# Trimmed and high quality raw reads assembled (n)	670,242,336	358,527,278
# Assembled contigs (n)	144,037	224,735
# Assembled contigs after contamination removal (n)	128,999	187.245
# Assembled “unigenes” (n)	72,165	94,884
# Predicted ORFs (n)	17,973	19,440
Average length (bp)	1,013.05	854.87
Median length (bp)	526	457
Total assembled bases (bp)	130,682,421	160,070,295
N50 (bp)	1,827	1,490
GC content for the entire assembly (%)	28.19	30.31
# KO terms	5,947	6,477

**Figure 3:**
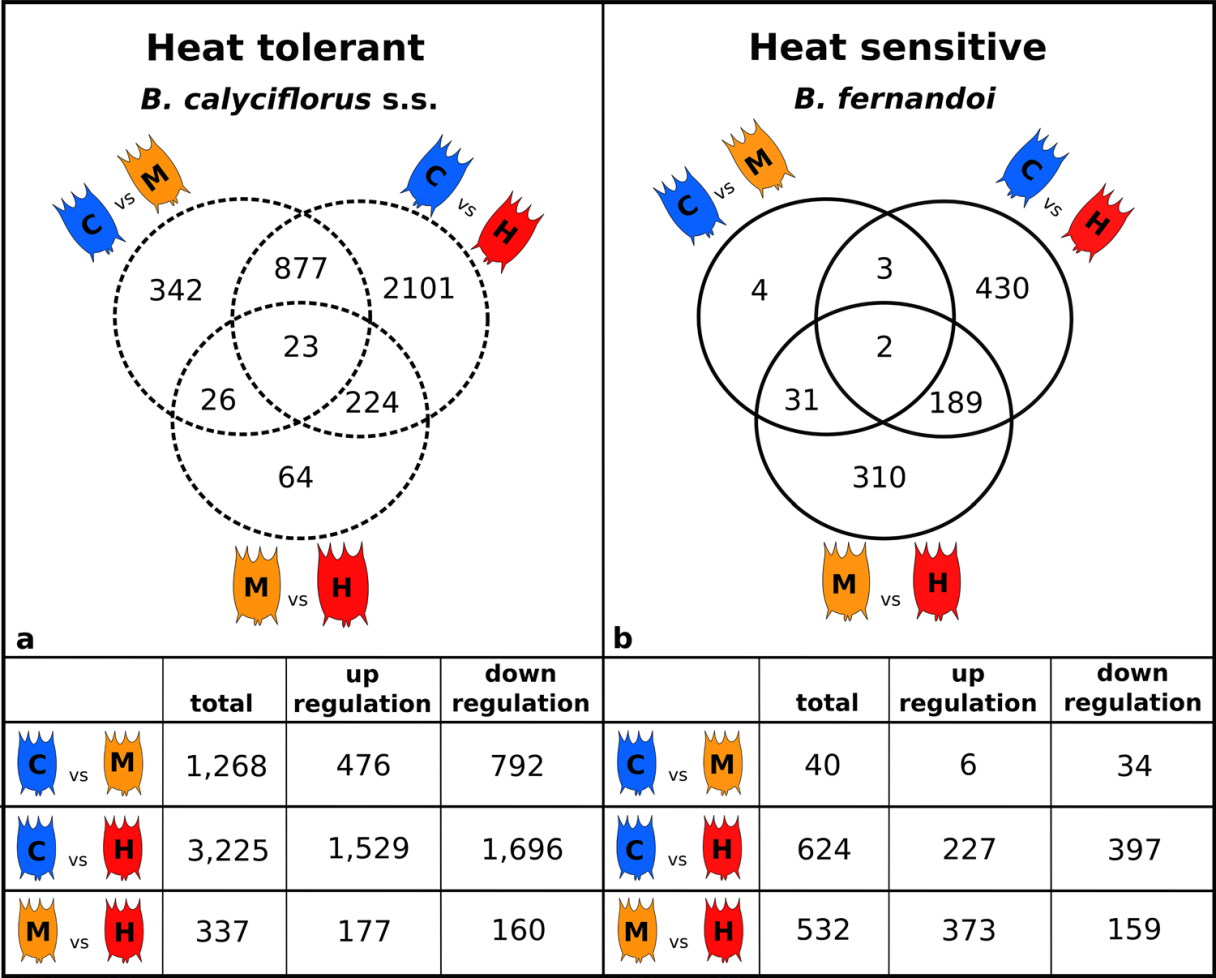
Venn diagrams showing the number of differentially expressed genes in pairwise temperature comparisons as determine by the DESeq2 analyses (FDR = 0.05) for the heat-tolerant *B. calyciflorus* s.s. (**a**) and the heat-sensitive *B. fernandoi* (**b**). Blue color represents control treatment (C), orange color represents mild heat treatment (M), and red color represents high heat treatment (H). Up- and down- regulation is defined from the perspective of the higher temperature over the lower one. (Figure made in Inkscape v.1.0; https://inkscape.org/).

We first examined the expression data for temperature-dependent responses in each species by a two-dimensional plot of the first two principal components of a PCA. This analysis showed temperature-dependent separation of replicates for the heat-tolerant, *B. calyciflorus* s.s.. This separation was less clear for the heat-sensitive species, in which replicates from 20 °C and 23 °C clustered together, while all samples treated at 26 °C formed a separate cluster (Supplementary Fig. 2). Overall, we found a greater number of differentially expressed genes (DEGs) in the heat-tolerant species than the heat-sensitive. In both species we captured the largest number of DEGs in pairwise comparison control vs*.* high heat (Fig. 3A,B). Further, in the heat-tolerant species, we captured a greater number of genes differentially expressed between control and mild heat (1,268) than between mild and high heat (337). In contrast, in the heat-sensitive species we found the opposite pattern, i.e., 40 DEGs between control and mild heat and 532 DEGs between mild and high heat (Fig. 3A,B).

We focused on pairwise comparison of mild vs. high heat in both species as we had evidence that there is a strong effect of temperature on population growth rate in this transition. In *B. calyciflorus* s.s., among the 337 genes differentially expressed between mild (26 °C) and high (32 °C) heat, were genes encoding for RNA polymerases (upregulated with heat), histone proteins (up- or downregulated) and *N*-acetyltranferases (upregulated with heat; Supplementary Fig. 3A). In heat-sensitive *B. fernandoi*, among the 532 differentially expressed genes between mild (23 °C) and high (26 °C) heat, we found genes encoding for several histone (H3 and H4) methyltranferase proteins (all up-regulated with heat; Supplementary Fig. 3B).

We further focused on genes that are differentially expressed in all the three pairwise comparisons. These genes are considered the most responsive to temperature, exhibiting either up- or down-regulation along with temperature increase. In the heat tolerant *B calyciflorus* s.s., there were 23 genes differentially expressed in all pairwise comparisons. Among these, genes encoding for ribosomal proteins and glutathione S-transferase were up-regulated at lower temperatures, while proteases related genes were up-regulated at higher temperatures (Supplementary Fig. 4A). In the heat-sensitive *B. fernandoi,* there were two genes that were differentially expressed in all pairwise comparisons. These genes encode for E3 ubiquitin-ligase and a mediator of RNA polymerase and were both up-regulated at higher temperatures (Supplementary Fig. 4B).

### Cross-temperature differentially expressed genes in heat shock response

**Figure 4:**
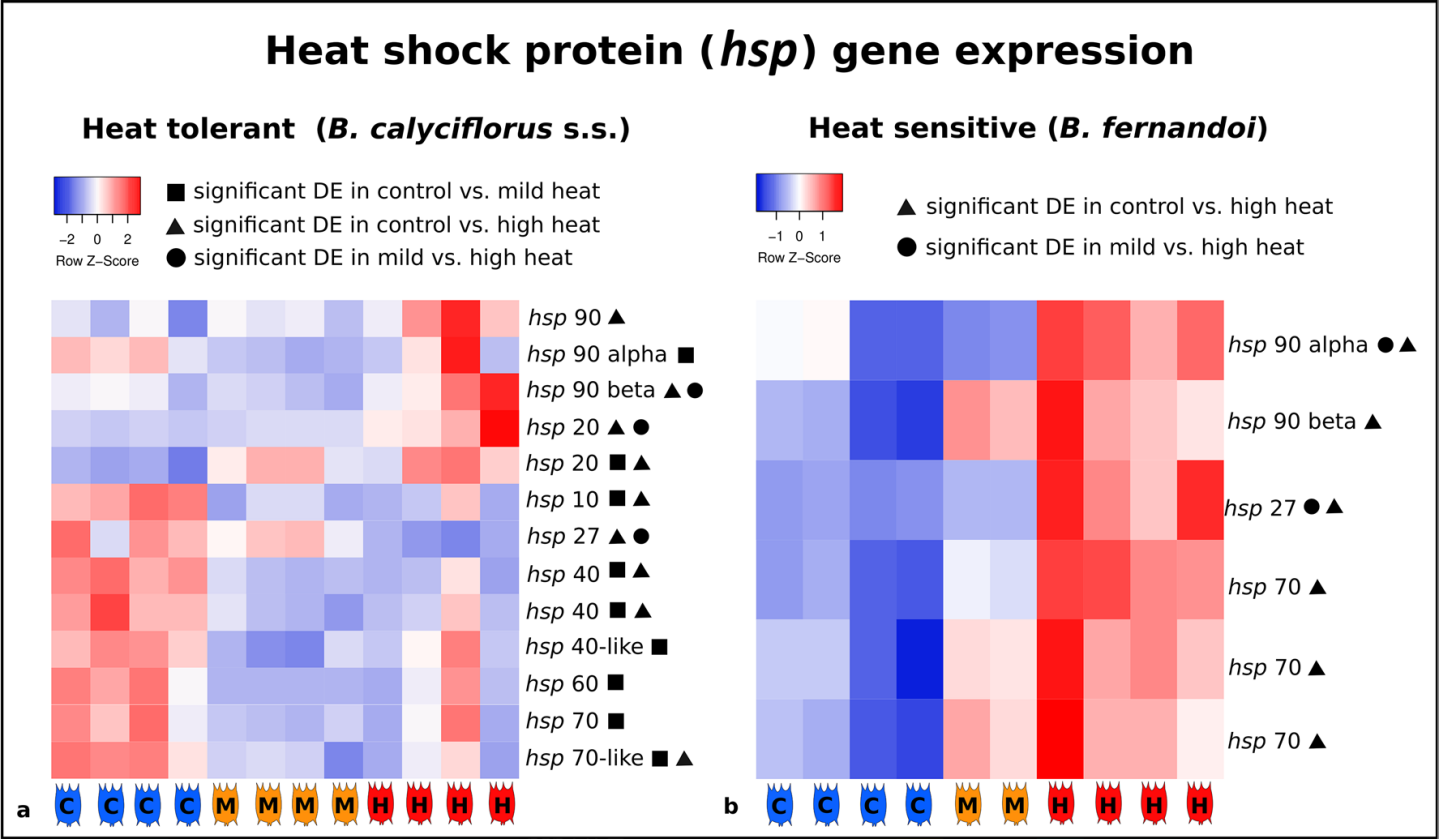
Heat map using normalized counts to show patterns of expression in *hsp* genes for the heat-tolerant *B. calyciflorus* s.s. (**a**), and the heat-sensitive *B. fernandoi* (**b**). Blue color (C) represents control treatment, orange color (M) represents mild heat treatment, and red color (H) represents high heat treatment. The symbols indicate the pairwise comparison in which differential gene expression (DE) was significant (p < 0.05). The normalized counts (relative expression normalized with DESeq2) were used for the heat maps and the color key represents a spectrum of lowest gene expression (blue) to highest gene expression (red). (Figure produced by the Heatmapper program^60^).

We examined patterns of expression in *hsp* genes to evaluate their specific contribution to the heat shock response (HSR) in both species. In both species, we found a significant down-regulation of heat shock protein genes at temperatures where population growth rate was maximized (Figs. 1B, 4A,B). In general, genes encoding for *hsp* had higher expression at the lowest temperature treatment (20 °C) for the heat-tolerant, *B. calyciflorus* s.s. and at the highest temperature for the heat-sensitive, *B. fernandoi.* Genes encoding for *hsp*27 and *hsp*70 followed this pattern. Genes encoding for *hsp*10, *hsp*40, and *hsp*60 were differentially expressed only in *B. calyciflorus* s.s. where they followed the same pattern. Genes encoding for *hsp90* beta were up-regulated under the highest imposed temperature regime in both species. In *B. calyciflorus* s.s., genes encoding for *hsp20* were also up-regulated under the highest imposed temperature (32 °C), following the induction pattern of *hsp90* beta (Fig. 4A).

### Cross-temperature differential KEGG pathway expression

**Figure 5:**
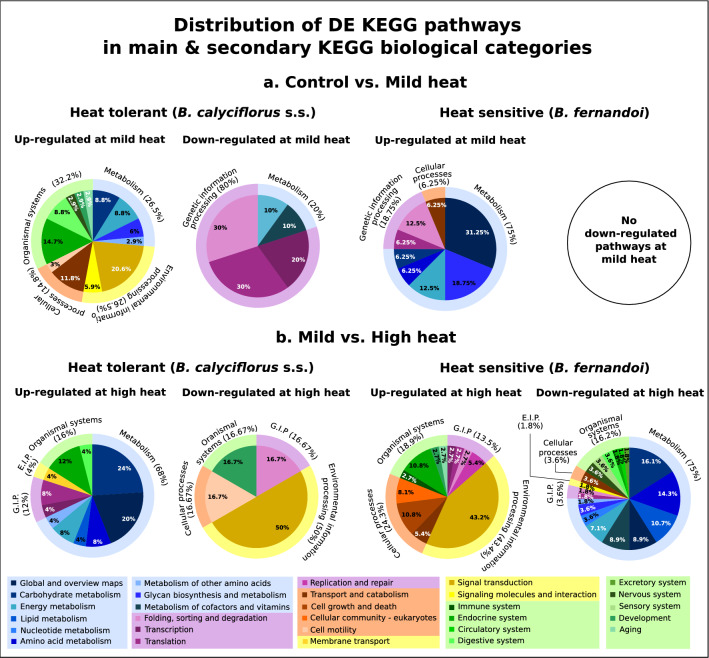
Distribution of differential expressed (DE) KEGG pathways (FDR = 0.05) in main and secondary KEGG biological categories in control vs. mild heat exposure (**a**), and in mild vs. high heat exposure (**b**). Left side represent changes captured for the heat tolerant, *B. calyciflorus* s.s. while right side depicts changes in the heat-sensitive *B. fernandoi*. (Figure made in Inkscape v.1.0; https://inkscape.org/).

To capture shared and contrasting patterns at the level of gene-pathways, we examined whole KEGG pathway expression in two pairwise comparisons, control vs. mild heat and mild vs. high heat, as both of these comparisons represent a stepwise temperature induction. Overall, mild heat resulted in down-regulation of pathways related to genetic information processing in heat-tolerant, *B. calyciflorus* s.s., and up-regulation of metabolism related pathways in the heat-sensitive, *B. fernandoi*. More specifically, pathways “ribosome”, “proteasome”, and “oxidative phosphorylation” were down-regulated under mild heat exposure for *B. calyciflorus* s.s. and up-regulated for *B. fernandoi* (Fig. 5A, Supplementary Figs. 5, 6). In general, high heat caused up-regulation of pathways related mainly to metabolism such as carbohydrate metabolism and lipid metabolism and down-regulation of pathways related to signal transduction for the heat-tolerant *B. calyciflorus* s.s. (Fig. 5B, Supplementary Fig. 7). The opposite was observed for the heat-sensitive, *B fernandoi*, in which pathways related to signal transduction were up-regulated, while pathways related to carbohydrate and lipid metabolism were down-regulation under high heat. Genes involved in meiosis pathway were also up-regulated under high heat, while genes involved in “ribosome” pathway were down-regulated for this species (Fig. 5B, Supplementary Fig. 8).

### Cross-species co-expression among groups of genes

**Figure 6:**
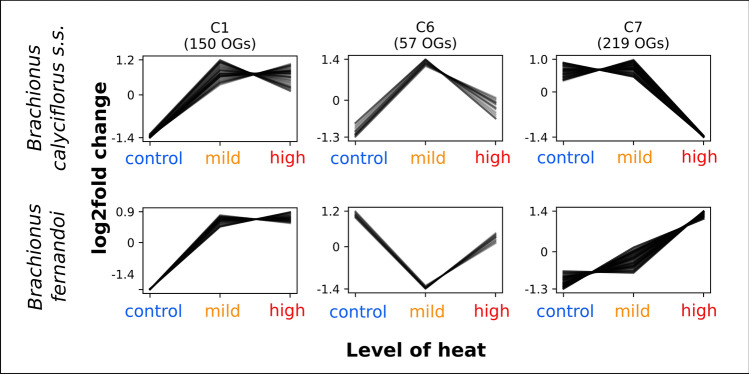
Shared and contrasting co-expression patterns of gene orthogroups (OGs) between heat-tolerant and heat-sensitive *Brachionus* species. C1, C6, C7 represent the numbers of clusters produced by Clust program^69^. The total number and co-expression pattern of all clusters are given in Supplementary Fig. 9.

To capture similar or contrasting patterns of expression between the two species, we searched for clusters of orthologous genes exhibiting co-expression. Analysis with Clust revealed the presence of 8 such gene groups/clusters (Supplementary Fig. 9). We have focused on just three of these because their patterns appear biologically relevant. Gene cluster C1 contained 150 orthogroups exhibiting the same expression pattern in both species, i.e., they were up-regulated with increasing temperature (Fig. 6). Five KEGG pathways were significantly enriched in the C1 group (p < 0.05, FDR = 0.05), among them genes belonging to core metabolic pathways (KEGG pathway: 01100). Comparing these data to the differentially expressed gene dataset, we found in total 3 genes (phosphatidylinositol-glycan-specific phospholipase D, acidic mammalian chitinase-like, transmembrane 144) that belonged to C1 cluster and were differentially expressed between control and mild heat exposure or between control and high heat exposure in both species.

Two clusters (C6 and C7) contained genes with opposing patterns of expression in the two species. In cluster C6, genes from the heat-tolerant *B. calyciflorus* s.s. had low expression at control conditions, were up-regulated at mild heat, and down-regulated again under high heat. In contrast, for *B. fernandoi*, genes in C6 cluster had higher expression under control conditions, were down-regulated at mild heat and up-regulated again at high heat. The longevity regulating pathway (KEGG pathway: 04212) was significantly enriched in C6 cluster (p < 0.05, FDR = 0.05). We found two genes that were differentially expressed in both species (BAI1-associated protein, uncharacterized protein LOC105328640) and at the same time co-expressed in this cluster. In cluster C7, for *B. calyciflorus* s.s. gene expression was high under control and mild heat conditions and down-regulated at high heat. However, for the heat-sensitive *B. fernandoi*, gene expression was low for control and mild heat conditions and up-regulated under high heat. In this cluster, a total number of 12 pathways were significantly enriched (p < 0.05, FDR = 0.05) among which pathways involved in signal transduction, nervous and endocrine systems, and replication and repair. Comparing to the differentially expressed gene dataset, we found 24 genes being differentially expressed in at least one pairwise comparison and at the same time belonging to C1 cluster for *B. calyciflorus* s.s. For the species *B. fernandoi*, 8 genes met the above two criteria. In total one gene (not annotated to any known protein) belonged to C7 cluster and was differentially expressed in mild vs. high heat for both species.

## Discussion

In response to stressful environmental conditions, an immediate cellular stress response is activated. Prolonged exposure to these environmental stimuli can additionally initiate a stressor-specific secondary response to re-establish homeostasis^70^. In contrast to the immediate response, which is triggered mainly by macromolecular damage or the generation of oxidative stress, the homeostasis response is triggered by stressor-specific sensors that identify changes, in particular environmental variables^70,71^. Studies from copepods, oysters and corals reveal that short-term temperature stress and long-term temperature stress response might involve different genes/pathways since long-term response requires a significant change of expression for many genes to ensure cellular homeostasis^28,72,73^. However, all of the above studies reported a significant overlap in expression responses to short- and long-term heat stress.

In the present study we combined life history and gene expression data to test variation in the temperature-dependent responses of species belonging to the recently resolved *B. calyciflorus* species complex^20^ with documented differences in short-term heat tolerance (heat-tolerant vs. heat-sensitive species)^23^. Our phenotypic data represent long-term adaptation to temperature, while the RNA-seq data represent short-term temperature responses. Nonetheless, we observed a transcriptomic response that was largely consistent with phenotypic data from life history experiments. This has revealed both shared and species-specific patterns in gene expression in response to heat and identified key functional pathways associated with temperature adaptation in these species. Our study demonstrates the power of testing the transcriptomic response of an organism to an environmental stressor by combining transcriptome with phenotypic data.

### Temperature-dependent life history responses in heat-tolerant vs. heat-sensitive *Brachionus* species

Rotifers living in temporally variable habitats are exposed to frequent changes in their environment that may impact their life history. In both species we found a profound effect of temperature on survival, with increasing temperature reducing their life span. Our results corroborate studies in other rotifer taxa that showed a profound effect of temperature on survival^22,74–77^. Due to their ectothermic nature, rotifer body temperature increases with increasing ambient temperature, which accelerates metabolic rates. Given unlimited food resources, juvenile and egg development are accelerated up to a certain critical temperature limit^78^. Fecundity, on the other hand did not follow the same pattern. Fecundity was significantly reduced only at high heat exposure (*B. calyciflorus* s.s., 32 °C; *B. fernandoi,* 26 °C), suggesting that fecundity is maintained across variable temperatures in both species, up to a limit above which a robust response cannot be maintained.

Population growth rate (*r*) is considered a proxy to evaluate environmental specializations and stress response, representing the ability of rotifers to grow in a particular environment^78–81^. Previous work in the rotifer complex of *Brachionus plicatilis* used population growth as a proxy to evaluate the salinity constrains between sibling species and found that—while species tolerated a wide range of salinities—their population growth rates responded differentially to this environmental factor^81–83^. It was hence suggested that optimal growth rate indicates adaptation to the respective environmental conditions. Consequently, environmentally triggered growth rate variation among species implies specializations, which facilitate dominance of the respective species in different periods of the year and makes sympatric co-occurrence possible^15^. In rotifers, broad temperature tolerance has been found^7,17,22^ which might reflect an adaptation to temperature fluctuations occurring in aquatic habitats. Both *B. calyciflorus* s.s. and *B. fernandoi* experience temperature fluctuations in their natural habitats and can survive a broad range of temperatures, however, their densities vary considerably, relative to ambient temperature^7,17,22^. High densities of *B. calyciflorus* s.s. have been reported in the summer up to 32 °C, while high densities of *B. fernandoi* have been reported during spring and winter even down to 4 °C^7,17^. We have shown that although both species can survive a relatively wide range of temperatures, both their population growth rate and expression of representative heat-stress genes is markedly different. Our results further corroborate that these species are specialized in their temperature tolerance, which might translate into habitat specializations and/or seasonal successions.

Differences in life histories between sibling species of the *B. calyciflorus* species complex have been found in response to competition and/or predation risk^84^. A recent study^85^ performed under stable food and temperature conditions (24 °C) showed differences in life history traits such as egg and juvenile developmental times, and egg production between these species. As a corollary, they demonstrated that observed differences are consistent across tested clones within species, i.e., they really represent differentiation between the species. According to our fitness results, our two species have evolved different strategies to respond to increased temperature, with *B. calyciflorus* adopting a life strategy of high population growth and low survival, as opposed to *B. fernandoi* with a strategy of low population growth and high survival. Our findings indicate that life histories of these two sibling species are differentially adapted, supporting the idea that the species are ecologically diverged and specialized for different environmental conditions, in particular with regard to temperature.

### Heat shock response in heat-tolerant vs. heat-sensitive *Brachionus* species

Heat shock response, which involves the induction of heat shock protein (*hsp*) genes, is a well known and evolutionary conserved mechanism present in both prokaryotes and eukaryotes^40^. Induction of *hsps* has been connected to several stress conditions such as exposure to extreme temperatures, heavy metals, pathogens, and osmotic stress^39^. In the present study, expression of *hsp* related genes mirrored measured changes in the population growth rate across a temperature gradient. Population growth rate was low when *hsp* genes were up-regulated. This pattern was consistent in both species, showing that *hsp* genes are indeed part of a species’ stress response, when environmental conditions (here, temperature) are outside the ‘comfort zone’ for optimal growth.

Closely related species differentially adapted to cold vs. warm habitats have also been found to express *hsp* genes differently in other aquatic organisms such as the amphipod *Eulimnogammarus.* In these amphipods, species originated from a cold habitat when exposed to heat, up-regulated *hsp* genes at lower temperatures compared to species from a warmer habitat^86^. In the present study, closely related *Brachionus* species with differences in their ability to tolerate heat have been found to express *hsp* genes differently. More specifically, *hsp* genes were induced outside the temperature of optimal growth: in the heat-sensitive *B. fernandoi, hsp* genes were induced by heat, while in heat-tolerant *B. calyciflorus* s.s. the majority of *hsp* genes were induced at the lower end of temperature exposure (20 °C), indicating that 20 °C may already be cold stress for the heat-tolerant species.

Proteins of the *hsp90* family serve to increase the available chaperons in the cells in order to recover from cellular stress and maintain structural integrity at high temperatures. In contrast to other *hsp* genes, their expression patterns supported a specific involvement in heat response, as genes encoding for *hsp90* were up-regulated towards the higher temperature regime in both species. The expression of *hsp90* gene has also been found to be temperature dependent in other aquatic organisms, including copepods and oysters^87–89^. This suggests that induction of *hsp90* gene along with heat might be a common mechanism in aquatic organisms. In *B. calyciflorus* s.s., genes encoding *hsp20* were also induced by high heat. Up-regulation of *hsp20* genes has been reported previously from other *Brachionus* species and copepods as a response to elevated temperatures^36,90^. Transformed bacteria (*Escherichia coli*) expressing the *Brachionus hsp20* (*Br-hsps20*) gene had a 100 fold increase in survival compared to the non transformed ones under high heat-stress, indicating that *Br-hsp20* specifically contributes to increased thermal tolerance^36^. Up-regulation of *hsp20* genes was found to also increase resistance to oxidative stress^36^. It is possible that an increase in expression of *hsp20* reflects a cellular defense mechanism in response to different stressors that might be common among *Brachionus* species.

A reversed expression pattern between our two species was found in genes encoding for *hsp70* and *hsp27*. In heat-tolerant *B. calyciflorus* s.s., genes encoding for *hsp70* were significantly up-regulated towards the lower temperature, while in *B. fernandoi* towards the higher temperature. It seems that temperatures such as 20 °C might constitute cold stress for warm adapted species and a stress response might be initiated under these conditions. In *B. calyciflorus* s.s., genes encoding for *hsp40* and *hsp60* followed the same induction pattern as *hsp70* genes, pointing towards a common mechanism regulating the expression of these three genes as reported also in *B. manjavacas*^37^. A synergistic relationship has been reported among *hsp40* and *hsp70* proteins, as *hsp40* regulates the ATPase activity of *hsp70*^91^. These genes were induced in *B. manjavacas* with increasing heat^37^. Apparently, these genes are induced under any condition that constitutes temperature stress for a particular *Brachionus* species.

### Metabolism response in heat-tolerant vs. heat-sensitive *Brachionus* species

Metabolism in ectotherms is inextricably linked to environmental temperature and its rate is accelerated by temperature increase. Genes belonging to core metabolic pathways had, in general, the same expression pattern in both the heat-tolerant *B. calyciflorus* s.s. and the heat sensitive *B. fernandoi,* showing an increased expression with temperature induction. However, there were significant differences between the species in genes involved in oxidative phosphorylation, lipid metabolism, and carbohydrate metabolism.

Oxidative stress is related to the production of toxic compounds that are called reactive oxygen species (ROS) which contribute to cellular damage and cause cellular response by modifying proteins and nucleic acids^92^. Exposure of ectothermic organisms to elevated temperatures accelerates mitochondrial respiration and potentially increases ROS formation^93,94^. ROS formation blocks heat shock response and refolding activity under heat stress, thereby leading to increasing cellular stress and ultimately heat sensitivity^95^. Genes related to oxidative stress were significantly up-regulated over temperature increase in heat-sensitive *B. fernandoi.* In contrast, these genes were significantly down-regulated over temperature increase in the heat-tolerant *B. calysiflorus* s.s.. Here, oxidative stress response was induced at the lowest temperature tested (20 °C). Among the differentially expressed genes were NADH dehydrogenase and glutathione S-transferase (*GST*). For the *B. calyciflorus* s.s., a significant induction (7 × fold change) of NADH dehydrogonase has been reported after sustained cold stress on 14 °C for 30 days^51^. Transcriptional regulation of *GST* genes also differed in two congeneric copepod species of the genus *Tigriopus*. For the species *T. japonicus,GST* genes were significantly down-regulated in response to temperature elevation up to 35 °C^96^. In the Pacific oyster, both of the above genes were down-regulated, dependent on the duration of heat exposure^89^. Furthermore, expression of the *GST *genes was either up- or down- regulated dependent on temperature intensity and duration of exposure in *Brachionus plicatilis*^34^. Transformed bacteria (*Escherichia coli*) expressing the *BrachionusGST* zeta gene were significantly protected against the oxidative stress induced by metals such as mercury and cadmium^97^. This indicates that repression of oxidation stress likely acts as a protective mechanism of the cells and potentially enhances heat tolerance^89^.

Lipid and carbohydrate metabolism are highly conserved processes that affect nearly all aspects of an organism’s biology. The consumed lipids and carbohydrates are broken down during digestion into fatty acids and simple sugars, providing the essentials to produce a wide range of metabolites that are required for development and survival. Genes related to lipid and carbohydrate metabolism were up-regulated from mild to high heat in heat-tolerant, *B. calyciflorus* s.s. and down-regulated in heat-sensitive *B. fernandoi*. Up-regulation with heat of genes related to carbohydrate metabolism has been identified after acute heat exposure in a teleost fish, *Gillichthys mirabilis*^98^ and in the Pacific oyster, *Crassostrea gigas*^99^. This suggests a need for rapid production of ATP under increasing temperatures. Apparently, *B. calyciflorus* s.s. has adapted to maintain its metabolism under high heat, while the heat-sensitive species *B. fernandoi* apparently shuts down costly metabolic processes (indicated by down-regulation of the majority of metabolic related pathways) in order to allocate available resources to survival.

### Ribosomal response in heat-tolerant vs. heat-sensitive *Brachionus* species

Ribosome biogenesis is a complex and energy-demanding process requiring coordination of ribosomal RNA (rRNA) and ribosomal protein production. Genes encoding for ribosomal proteins have been identified several times as a part of stress response and they have been either induced or suppressed upon temperature increase^87,89,100,101^. Ribosomal protein related genes were up-regulated towards the lower imposed temperature of 20 °C in the heat tolerant *B. calyciflorus* s.s., indicating again that 20 °C likely comprise stressful conditions for this species. In contrast, in the heat-sensitive *B. fernandoi*, ribosomal protein related genes were up-regulated under mild heat stress (23 °C), suggesting an increased translation capacity or a protection of ribosomal function through the addition or replacement of ribosomal proteins^72^. However, further temperature increase up to 26 °C, resulted in down-regulation of ribosomal related genes. This suppression of protein biosynthesis may reflect cellular homeostasis or an energy saving mechanism to cope with thermal stress, as protein metabolism consumes a large amount of ATP.

### Other molecular mechanisms of heat response in heat-tolerant and heat-sensitive *Brachionus* species

*Brachionus*, as most monogonont rotifers, have two reproductive modes, one asexual allowing for fast population growth and one sexual to promote recombination under unfavorable environmental conditions^102,103^. The sexual phase of reproduction is generally induced by environmental factors such as photoperiod, population density and food composition^102–104^. Rotifer species are capable of abandoning either the sexual or the asexual phase. Abandoning sexual reproduction is very rare in nature, however, it is a common phenomenon in clones that have been under laboratory cultivation over a long period of time and it relies on a recessive allele^105,106^. In *B. fernandoi*, increase of temperature resulted in significant up-regulation of genes related to meiosis, indicating that temperature exposure above 23 °C triggered sexual reproduction. In *B. calyciflorus* s.s., there was no significant up-regulation of meiosis-related genes, neither at high nor at low temperatures. Possible explanations are that this clone has lost the ability of sexual reproduction or that sexual reproduction is triggered by temperatures beyond the range tested here or stimuli other than temperature.

Epigenetic control on transcription can be achieved by many mechanisms, including DNA methylation or post-translational modifications to histone tails, including histone methylation and acetylation. It is known from genomic/transcriptomic studies of *B. manjavacas* and other rotifers that rotifers lack DNA methyltransferases (*Dnmt1*, *Dnmt3*) for epigenetic transcriptional regulation^31,56^. However, they do not lack the molecular machinery for post-translational regulation to histone tails, which play an important role in regulating gene expression. Histone tails are modified by enzymes called histone methyltransferases which catalyze the transfer of one, two or three methyl groups to lysine or arginine residues of histone protein tails^107^. Thus, there are two main types of histone methyltransferases, the lysine-targeting (e.g., histone-lysine N-methyltransferases) and the arginine-targeting (e.g., histone-arginine N-methyltransferases). The number of methyl groups attached in specific sites of histone tails and the amino acid which is methylated in histones determine the activation or suppression of gene transcription. In *B*. *fernandoi*, exposure to high temperatures resulted in up-regulation of histone (H3 and H4) methyltransferase genes. Histone H4K20 methyltransferase, catalyses a single methylation of lysine (20 residue) in histone H4, forming the dimethylated form. This modification has been related to silencing chromatin^108^. Silencing of chromatin might be related to translation suppression that we found for the heat-sensitive species under high heat exposure. In contrast, histone H3K4 trimethyltransferase catalyses a trimethylation of lysine (4 residue) in histone H3, ultimately generating a trimethylated form. This modification influences the binding of chromatin-associated proteins and in most cases the trimethylation of this position is associated with gene activation^109^. Transcriptional activation via trimethylation of H3K79 and H3K4 sites might be associated with a numerous environmental-information-processing pathways that were up-regulated in this species under high heat exposure.

## Conclusions

We found significantly different responses to heat between heat-tolerant and heat-sensitive *Brachionus* species. Transcriptomic responses were found to correlate with differences in fitness and especially differences in population growth, indicating underlying mechanisms of phenotypes’ responses to environmental change. Generally, the respective species upregulated metabolism/translation related genes under the temperature where their growth rate was high, while stress related (and—in one species—meiosis related) genes were expressed beyond the temperature regime optimal for growth. What had been historically considered the single species *B. calyciflorus* actually comprises several closely related rotifer species, which are differentially adapted to different environmental conditions (here, temperature). We have shown that this is driven by differing gene expression profiles and peak performance temperature. These differences allow them to occur in sympatry, but in different seasons. The genes found to be upregulated under heat stress might be targets of selection potentially contributing to the ecological divergence of the two species. Additionally, their expression profiles might be used as biomarkers to assess species vulnerability to environmental conditions and climate changes.

Our experimental setup aimed at minimizing the influence of factors other than temperature. As a general rule in ectotherms, temperature has a strong influence on developmental time by shortening time to maturation and generation time. By applying a short-term heat exposure of 4 h, we minimized a differential age-structure effect among the temperature treatments. Different developmental stages (i.e. juveniles, mature females with and without eggs, no mictic females nor males were observed) were pooled together randomly in all temperatures treatments. Admittedly, while the growth conditions prior to the heat exposure guaranteed substantial population growth rate and a mixture of all life stages, we could not control for exactly equal relative share of all life stages in the experimental treatments. This might have contributed minor variations in our observed expression levels regarding genes connected to oogenesis, mitosis, and cell cycle.

## Supplementary information

Supplementary Information 1.

Supplementary Information 2.

## Data Availability

All SRA files are available from the Gen Bank database (accession numbers: SRR10426055-76). All other data that support the findings of this study are included in online supplementary material.
