# Supplementary material for: Temperature-dependent life history and transcriptomic responses in heat-tolerant versus heat-sensitive Brachionus rotifers
Source: Sci Rep. 2020 Aug 6;10:13281. doi: 10.1038/s41598-020-70173-0 (PMC7411042; doi:10.1038/s41598-020-70173-0)
Supplement: Supplementary file 2 — Supplementary Information 2. [file 41598_2020_70173_MOESM2_ESM.docx]

### Temperature-dependent life history and transcriptomic responses in heat-tolerant versus heat-sensitive *Brachionus* rotifers

Sofia Paraskevopoulou, Alice B. Dennis, Guntram Weithoff, Ralph Tiedemann

**Supplementary Table 1.** Log-rank p-values for pairwise comparing the kaplan-Meier survival curves. Bcal stands for *B. calyciflorus* s.s. while Bfer for *B. fernandoi* . Numbers (20, 23, 26, 32) represent the tested temperatures.

|  | **Bcal20** | **Bcal23** | **Bcal26** | **Bcal32** | **Bfer20** | **Bfer23** | **Bfer26** |
| --- | --- | --- | --- | --- | --- | --- | --- |
| **Bcal20** | - |  |  |  |  |  |  |
| **Bcal23** | 0.137 | - |  |  |  |  |  |
| **Bcal26** | 0.014 | 0.067 | - |  |  |  |  |
| **Bcal32** | <0.001 | <0.001 | <0.001 | - |  |  |  |
| **Bfer20** | <0.001 | <0.001 | <0.001 | <0.001 | - |  |  |
| **Bfer23** | 0.007 | 0.008 | <0.001 | <0.001 | 0.001 | - |  |
| **Bfer26** | 0.002 | 0.032 | 0.189 | 0.02 | <0.001 | <0.001 | - |

**Supplementary Table 2.** Wilcox test p-values corrected with Bonferoni test for pairwise comparisons of fecundity data presented in Figure S1. Bcal stands for *B. calyciflorus* s.s. while Bfer for *B. fernandoi* . Numbers (20, 23, 26, 32) represent the tested temperatures.

|  | **Bcal20** | **Bcal23** | **Bcal26** | **Bcal32** | **Bfer20** | **Bfer23** | **Bfer26** |
| --- | --- | --- | --- | --- | --- | --- | --- |
| **Bcal20** | 1 |  |  |  |  |  |  |
| **Bcal23** | 1 | 1 |  |  |  |  |  |
| **Bcal26** | 1 | 1 | 1 |  |  |  |  |
| **Bcal32** | 0.00018 | 0.00138 | 0.00208 | 1 |  |  |  |
| **Bfer20** | 1 | 1 | 1 | 0.00202 | 1 |  |  |
| **Bfer23** | 1 | 0.57898 | 0.81126 | 0.07814 | 0.5128 | 1 |  |
| **Bfer26** | 2.6E-06 | 0.00058 | 0.00043 | 1 | 0.00094 | 0.00445 | 1 |

**Supplementary Table 3.** Number of reads per each library (replicate) before and after trimming for *B. calyciflorus* s.s. and *B. fernandoi.* Numbers (20, 23, 26, 32) represent the tested temperatures in which the data were produced.

| ***Brachionus calyciflorus*** **s.s.** | | | ***Brachionus fernandoi*** | | |
| --- | --- | --- | --- | --- | --- |
| **libraries** | **# Raw reads** | **# Reads after triming** | **libraries** | **# Raw reads** | **# Reads after triming** |
| T20_rep1 | 34,749,666 | 30,615,586 | T20_rep1 | 37,253,638 | 31,155,660 |
| T20_rep2 | 27,894,087 | 24,965,617 | T20_rep2 | 32,042,855 | 25,848,450 |
| T20_rep3 | 31,235,887 | 27,844,439 | T20_rep3 | 39,981,854 | 33,697,000 |
| T20_rep4 | 26,442,433 | 22,115,514 | T20_rep4 | 39,280,337 | 31,646,786 |
| T32_rep1 | 41,086,263 | 33,371,857 | T23_rep1 | 33,985,710 | 28,594,181 |
| T32_rep2 | 38,109,651 | 31,285,446 | T23_rep2 | 32,478,779 | 28,321,562 |
| T32_rep3 | 33,029,574 | 26,217,010 | T26_rep1 | 30,833,507 | 26,823,405 |
| T32_rep4 | 36,448,547 | 29,416,476 | T26_rep2 | 35,303,553 | 27,591,725 |
| T26_rep1 | 31,667,963 | 28,494,577 | T26_rep3 | 34,452,732 | 29,923,512 |
| T26_rep2 | 30,116,203 | 24,337,020 | T26_rep4 | 35,386,170 | 30,671,313 |
| T26_rep3 | 38,747,347 | 29,805,127 |  |  |  |
| T26_rep4 | 29,819,112 | 26,652,499 |  |  |  |


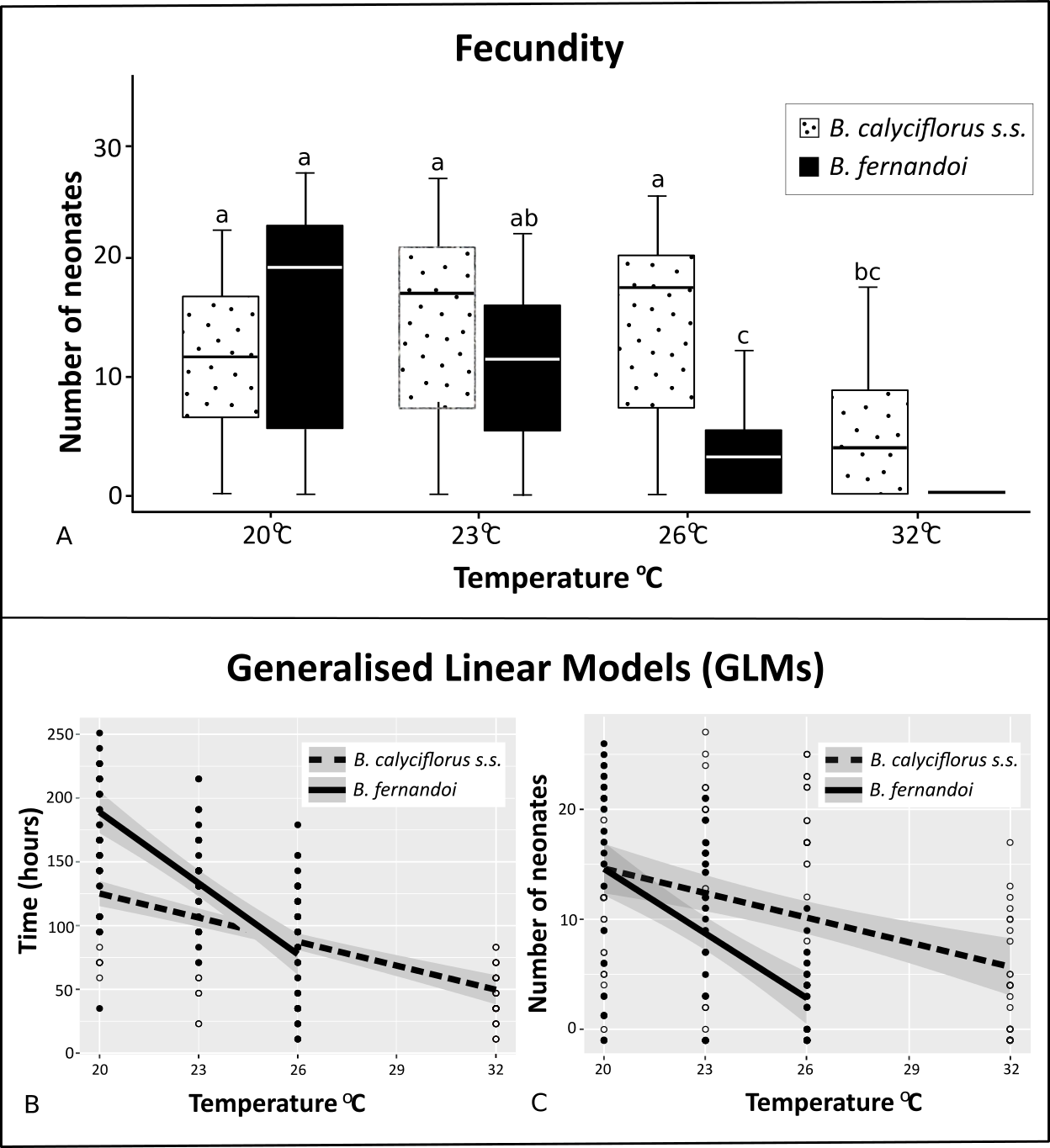


**Supplementary Figure 1**. (A) Box blot of fecundity for *B. calyciflorus* s.s. (black dots) and *B. fernandoi* (solid black) species in all tested temperature assays. Error bars indicate the standard deviation from the mean. Letters indicate pairwise Wilcox comparisons corrected with Bonferroni test. Different letters indicate significant differences, p<0.05. (B) Visualization of the GLMs for *B. calyciflorus* s.s. (dashed line) and *B. fernandoi* (solid line) of the survival time (in hours). (C) Visualization of the GLM model for *B. calyciflorus* s.s. (dashed line) and *B. fernandoi* (solid line) of the fecundity (number of neonates). (Figure produced by using R 3.4.1 [1]).


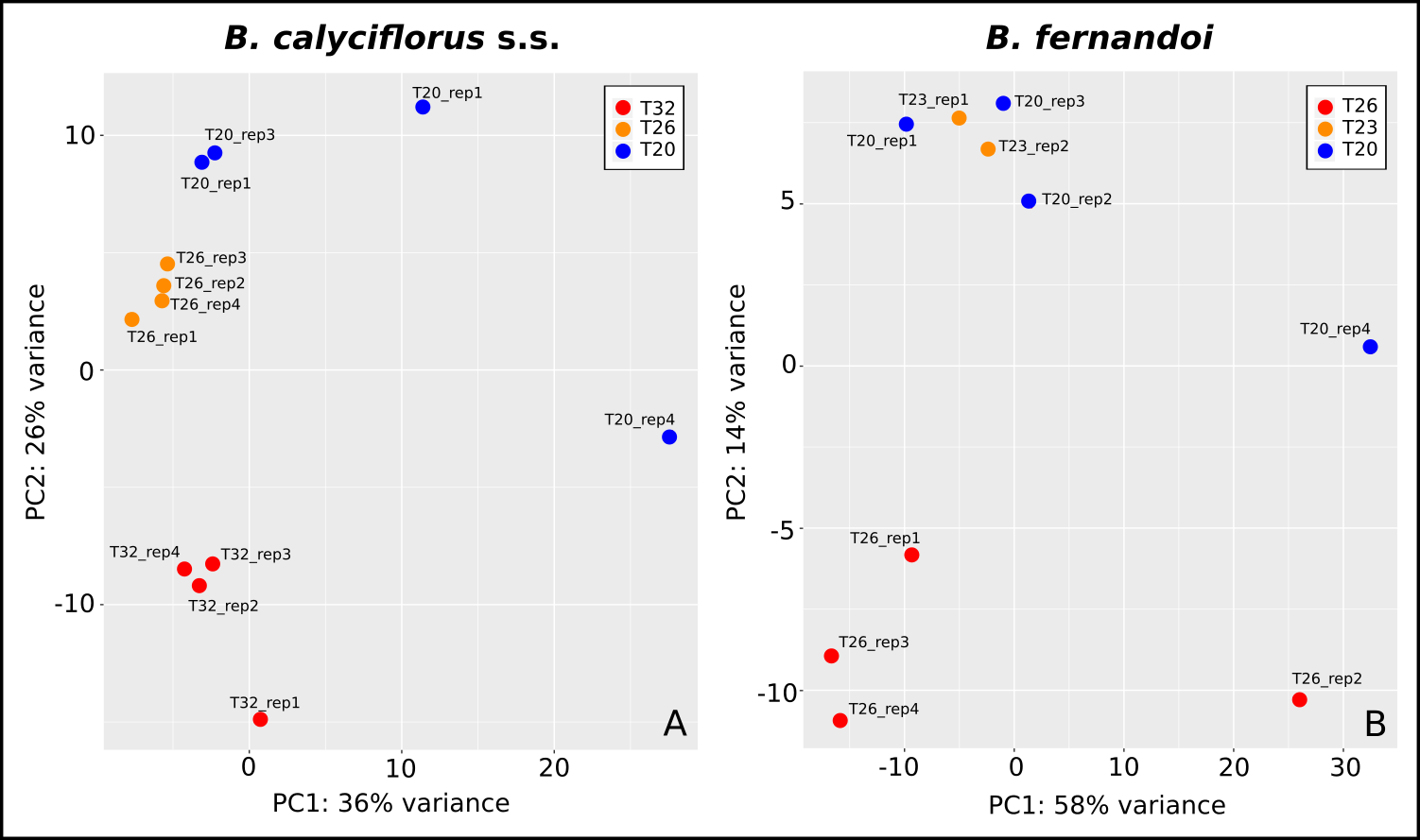


**Supplementary Figure 2**. PCA plot of the two first principal components of the total expression data of assembled genes for all species/treatment replicates for *B. calyciflorus* s.s. (A), and *B. fernandoi* (B)*.* (Figure produced by using R 3.4.1 [1]).


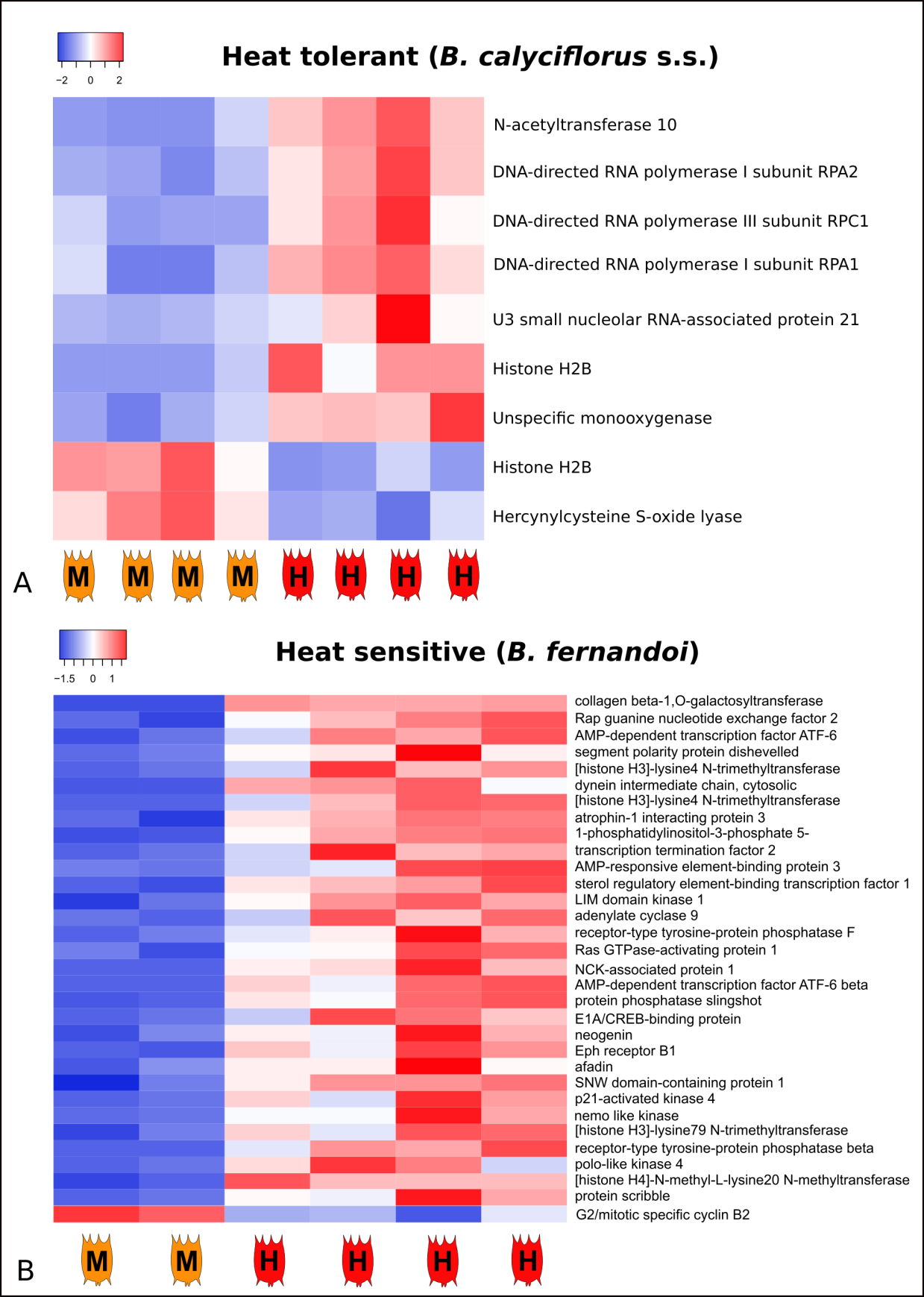


**Supplementary Figure 3.** Heat map showing the patterns of expression of differentially expressed genes (DEGs) present only in mild *vs.* high heat pairwise comparison for *B. calyciflorus* s.s. (A), and *B. fernandoi* (B). Only genes belonging to overrepresented KEGG pathways are presented here. Orange color (M) represents mild heat treatment and red color (H) represents high heat treatment. The normalized counts (relative expression normalized with DESeq2) were used for the heat maps and the color key represents a spectrum of lowest gene expression (blue) to highest gene expression (red). (Figure produced by using Heatmapper [2]).


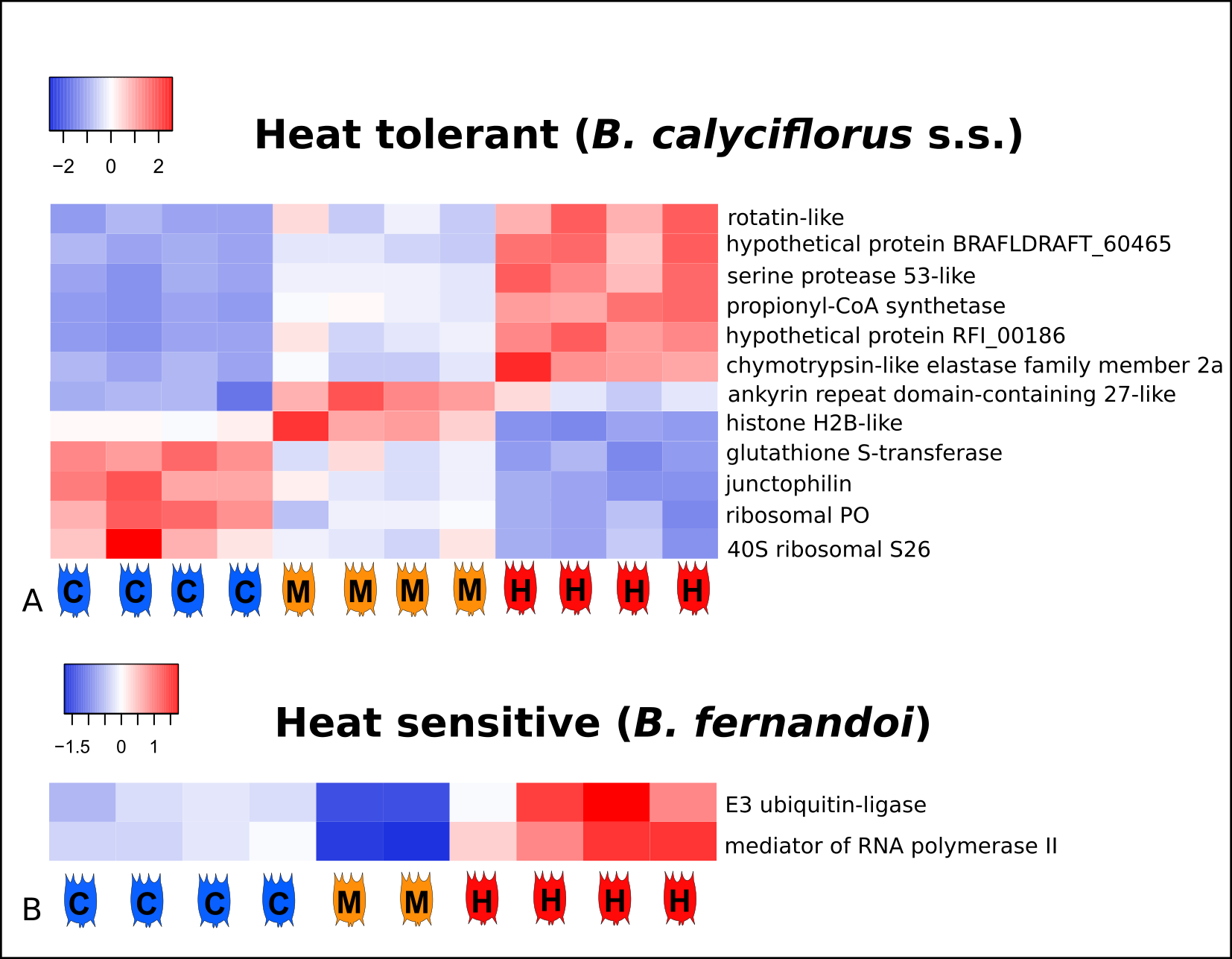


**Supplementary Figure 4**. Heat map showing the patterns of expression of differentially expressed genes (DEGs) present in all three pairwise comparisons in *B. calyciflorus* s.s. (A), and *B. fernandoi* (B). Blue color (C) represents control treatment, orange color (M) represents mild heat treatment, and red color (H) represents high heat treatment. The normalized counts (relative expression normalized with DESeq2) were used for the heat maps and the color key represents a spectrum of lowest gene expression (blue) to highest gene expression (red). (Figure produced by using Heatmapper [2]).


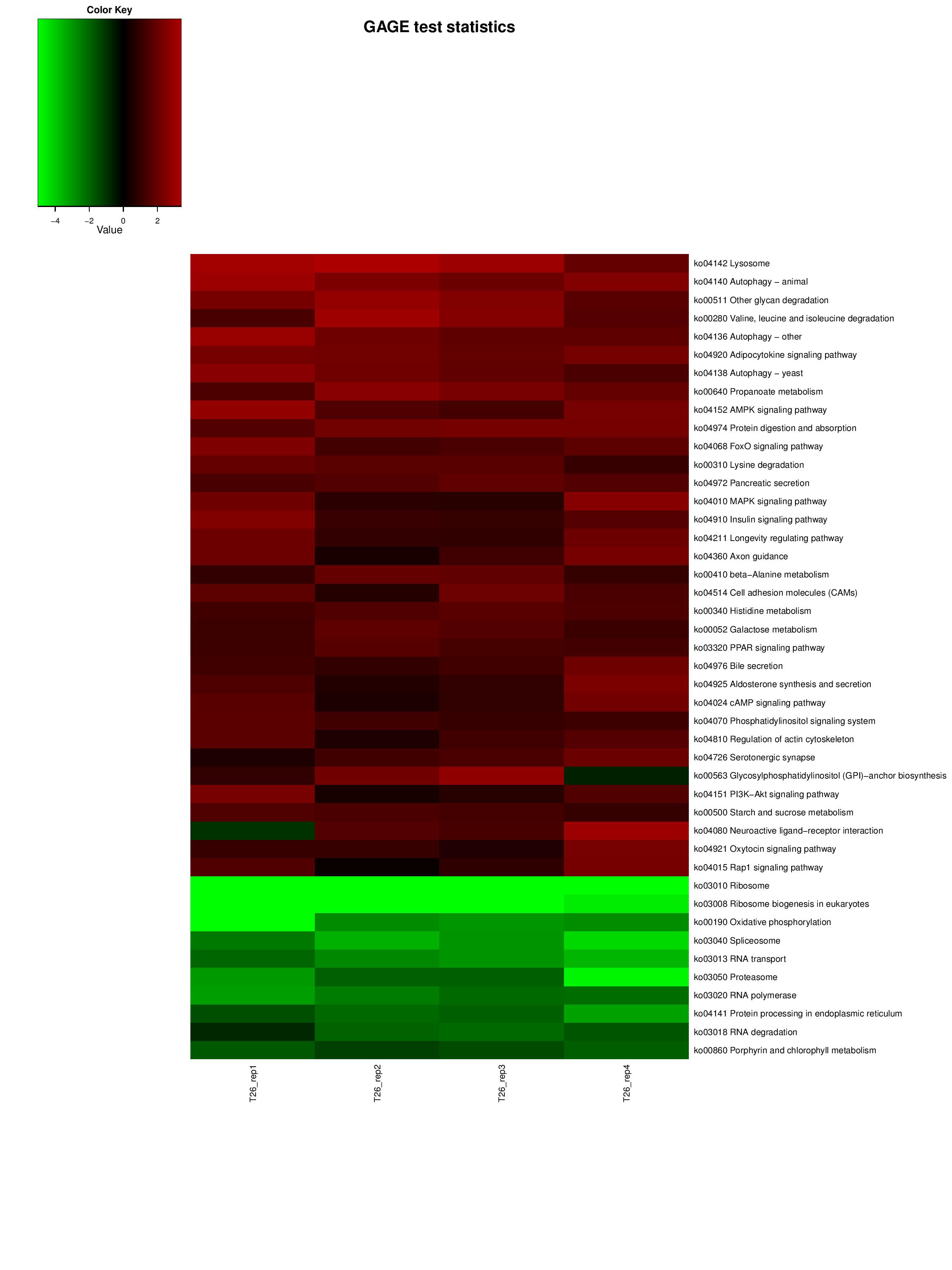

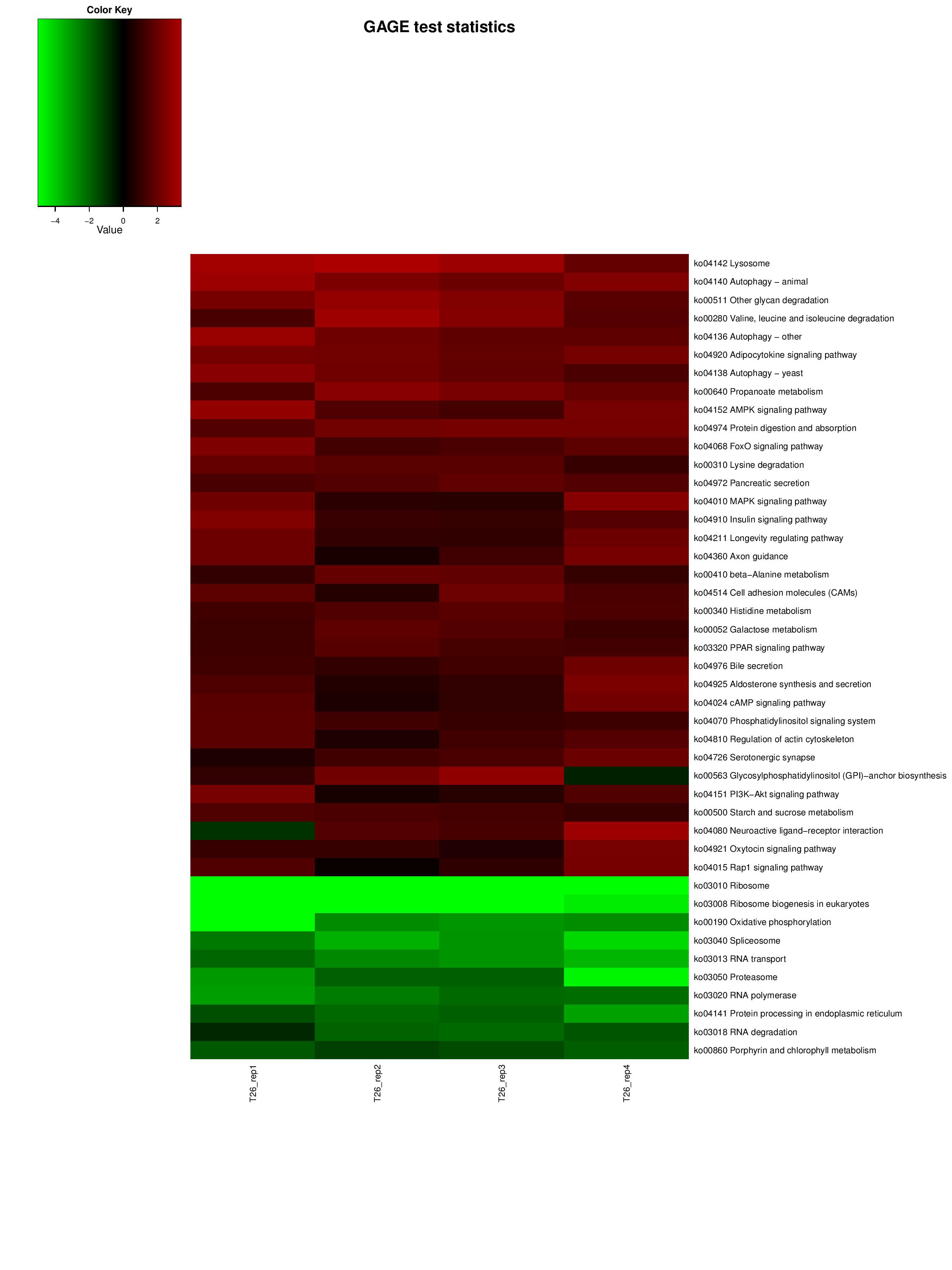
**Supplementary Figure 5.** Pathways up- and down-regulated in control *vs.* mild heat comparison for the species *B. calyciflorus* s.s.. Red and green color represents up- and down-regulation, respectively, at mild heat. Heat map is column normalized. (Figure produced by using R 3.4.1 [1]).


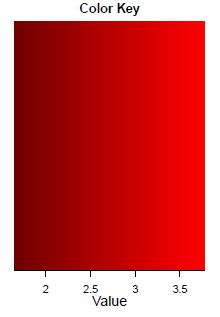

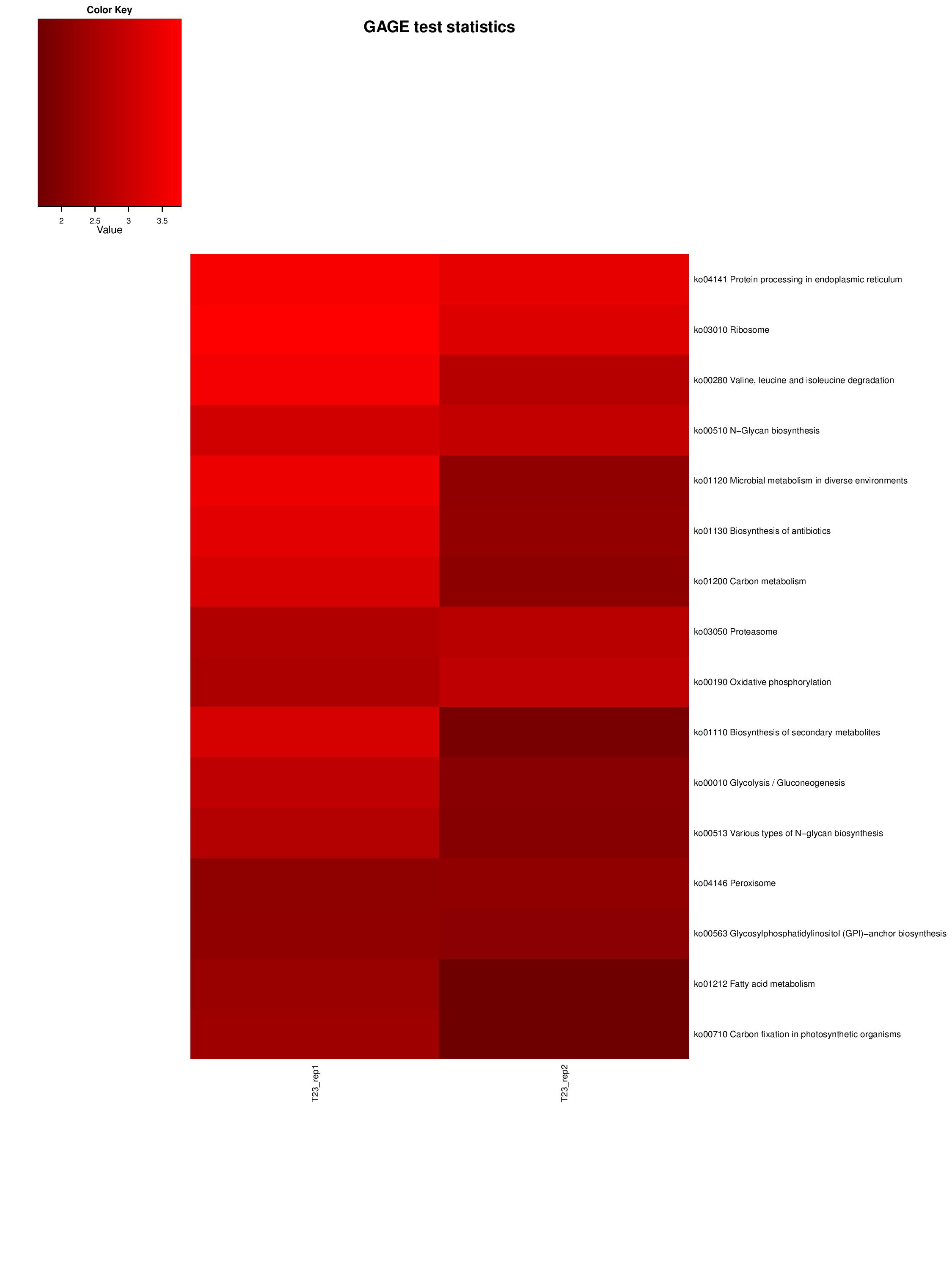
**Supplementary Figure** **6.** Pathways up-regulated in control *vs.* mild heat comparison for the species *B. fernandoi*. Red and green color represents up- and down-regulation respectively, at mild heat. Heat map is column normalized. (Figure produced by using R 3.4.1 [1]).


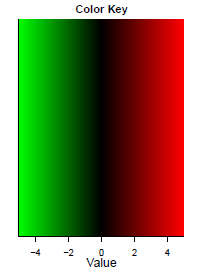


**
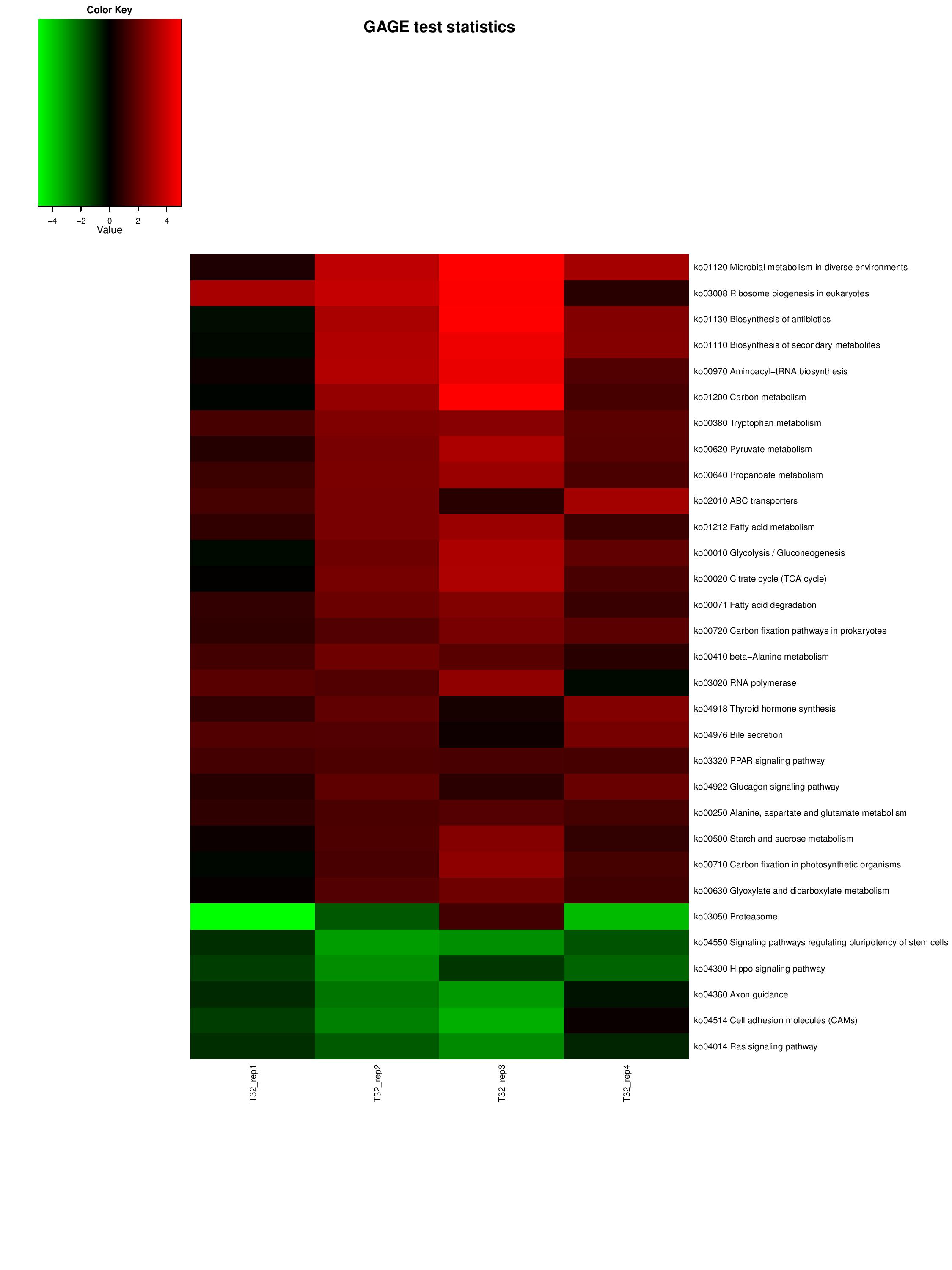
**

**Supplementary Figure** **7.** Pathways up- and down-regulated in mild *vs.* high heat comparison for the species *B. calyciflorus* s.s.. Red and green color represents up- and down-regulation respectively, at high heat. Heat map is column normalized. (Figure produced by using R 3.4.1 [1]).


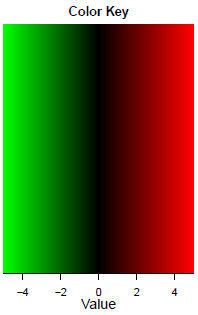

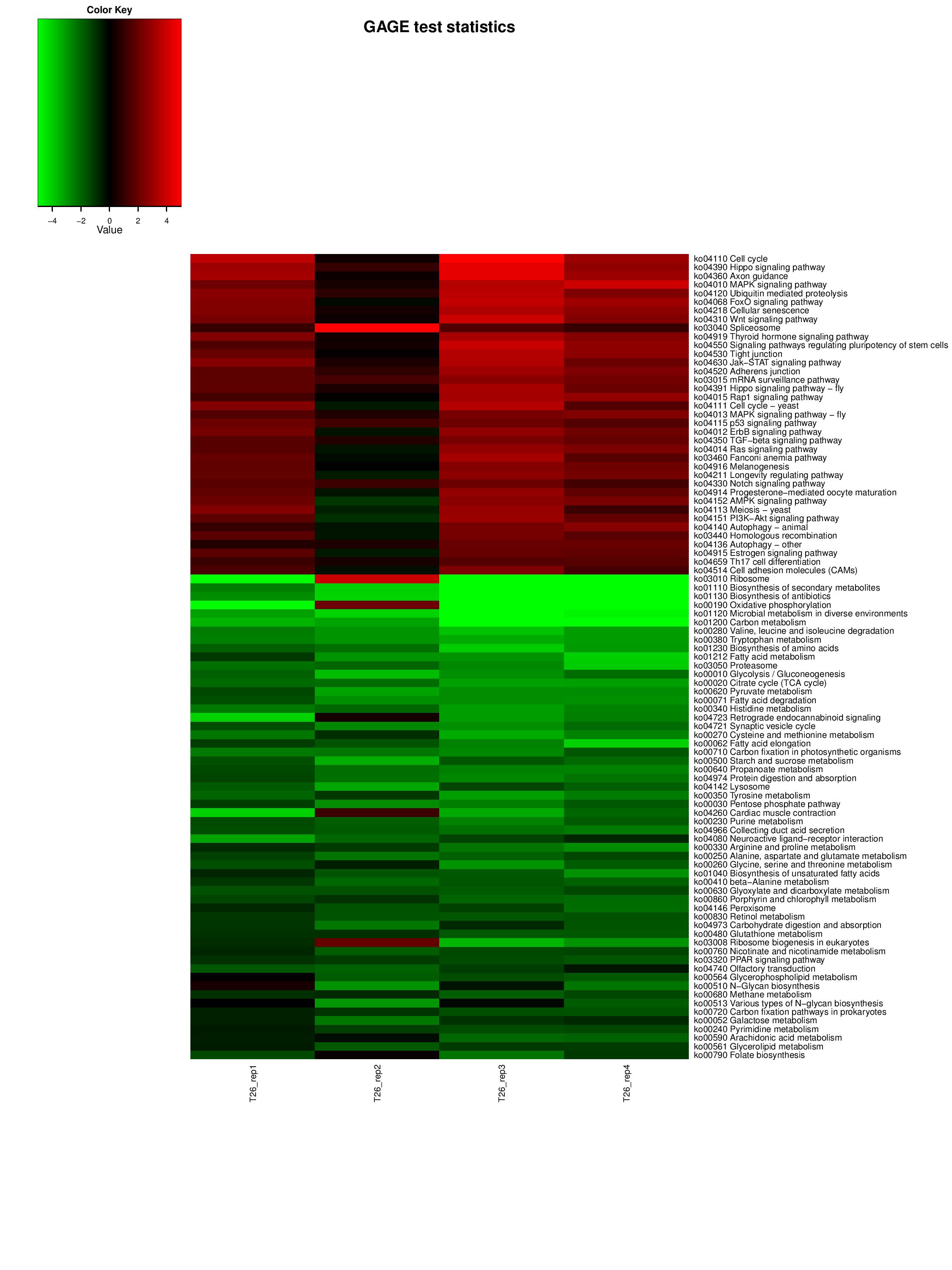


**Supplementary Figure** **8.** Pathways up- and down-regulated in mild *vs.* high heat comparison for the species *B. fernandoi*. Red and green color represents up- and down-regulation respectively, at high heat. Heat map is column normalized. (Figure produced by using R 3.4.1 [1]).


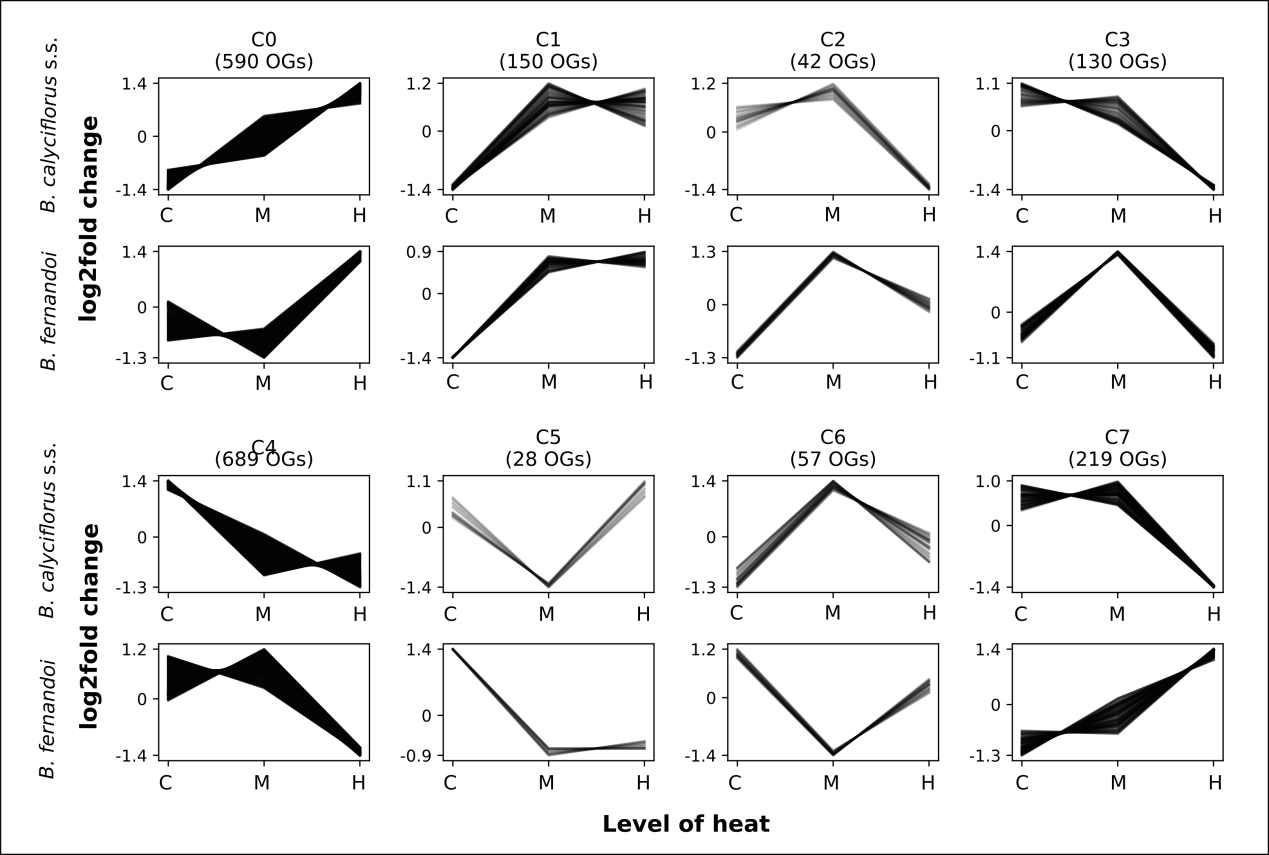


**Supplementary Figure** **9.** Co-expression patterns of gene orthogroups (OGs) between heat-tolerant (*B. calyciflorus* s.s.) and heat-sensitive (*B. fernandoi*) species. C0-C7 represent the number of clusters provided by the *Clust* program [3]. C: control treatment, M; mild heat treatment, H: high heat treatment.

**References**

1. R CoreTeam. *R: A language and environment for statistical computing.* R Foundation for Statistical Computing, Vienna, Austria http//:www.R-projest.org (2013).
2. Babicki, S. *et al.*  Heatmapper: web-enabled heat mapping for all. [*Nucleic Acids Res*. **44**, W147–W153; 10.1093/nar/gkw419](http://www.ncbi.nlm.nih.gov/pubmed/27190236) (2016).
3. Abu-Jamous, B. & Kelly, S. Clust: Automatic extraction of optimal co-expressed gene clusters from gene expression data. *Genome Biol.* **19,** 172; 10.1186/s13059-018-1536-8 (2018).
